# Functional Organization of a Multimodular Bacterial Chemosensory Apparatus

**DOI:** 10.1371/journal.pgen.1004164

**Published:** 2014-03-06

**Authors:** Audrey Moine, Rym Agrebi, Leon Espinosa, John R. Kirby, David R. Zusman, Tam Mignot, Emilia M. F. Mauriello

**Affiliations:** 1 Laboratoire de Chimie Bactérienne, Institut de Microbiologie de la Méditerranée, CNRS-Aix-Marseille University, Marseille, France; 2 Department of Microbiology, University of Iowa, Iowa City, Iowa, United States of America; 3 Department of Molecular and Cell Biology, University of California, Berkeley, Berkeley, California, United States of America; University of Geneva Medical School, Switzerland

## Abstract

Chemosensory systems (CSS) are complex regulatory pathways capable of perceiving external signals and translating them into different cellular behaviors such as motility and development. In the δ-proteobacterium *Myxococcus xanthus*, chemosensing allows groups of cells to orient themselves and aggregate into specialized multicellular biofilms termed fruiting bodies. *M. xanthus* contains eight predicted CSS and 21 chemoreceptors. In this work, we systematically deleted genes encoding components of each CSS and chemoreceptors and determined their effects on *M. xanthus* social behaviors. Then, to understand how the 21 chemoreceptors are distributed among the eight CSS, we examined their phylogenetic distribution, genomic organization and subcellular localization. We found that, *in vivo*, receptors belonging to the same phylogenetic group colocalize and interact with CSS components of the respective phylogenetic group. Finally, we identified a large chemosensory module formed by three interconnected CSS and multiple chemoreceptors and showed that complex behaviors such as cell group motility and biofilm formation require regulatory apparatus composed of multiple interconnected Che-like systems.

## Author Summary

*Myxococcus xanthus* is a social bacterium that exhibits a complex life cycle including biofilm formation, microbial predation and the formation of multicellular fruiting bodies. Genomic analyses indicate that *M. xanthus* produces an unusual number of chemosensory proteins: eight chemosensory systems (CSS) and 21 chemoreceptors, 13 of which are orphans located outside operons. In this paper we used genetic, phylogenetic and cell biology techniques to analyze the organization of the chemoreceptors and their functions in the regulation of *M. xanthus* social behaviors. Results indicate the existence of one large and three small chemosensory modules that occupy different positions within cells. This organization is consistent with *in vivo* protein interaction assays. Our analyses revealed the presence of a complex network of regulators that might integrate different stimuli to modulate bacterial social behaviors. Such networks might be conserved in other bacterial species with a life cycle of similar complexity and whose genome carries multiple CSS encoding operons.

## Introduction

Perceiving and responding to external stimuli allows living organisms to adapt to changes in their environment and thus enhance their survival fitness. Perception universally occurs through the aid of receptors coupled to signaling pathways that translate an initial signal into the appropriate cellular behaviors. Perception of stimuli in bacteria is largely mediated by one-component, two-component and chemosensory systems (CSS). CSS are modified two-component systems in which the histidine kinase, CheA, does not directly perceive the chemical signal [1]. Instead, this function is delegated to specialized chemoreceptors, known as Methyl-accepting Chemotaxis Proteins (MCPs) for the presence of a methyl-accepting domain in their C-terminal cytoplasmic region [2]. An adaptor protein, CheW, facilitates the interaction between the MCP and the CheA proteins. MCPs are methylated and demethylated on glutamate residues by a methyltransferase (CheR) and a methylesterase (CheB), respectively [2]. These enzymatic activities allow adaptation of the receptor to persistent stimuli [3]. The best-studied CSS are specialized for chemotaxis. In this case, the output response regulator CheY has the function of directly communicating with the flagellar motor proteins, FliM and FliN, in order to adjust the cell swimming behavior [4]. Interestingly, over the past years, many CSS have been identified that regulate behavioral responses other than taxis [5]. Examples are the *Myxococcus xanthus* Che3 system that regulates gene expression during development [6], the *Pseudomonas aeruginosa* Wsp system that regulates c-di-GMP production and biofilm formation [7] and the *Rhodospirillum centenum* Che3 system involved in cyst formation.

When multiple receptors mediate signal reception and stimulate kinase activity, the various signals must be integrated to generate a single response. For example, in the *E. coli* Che system that contains a single chemosensory pathway, five receptors of different ligand specificity signal to the same kinase, CheA [8], [9]. However, in bacteria with multiple chemosensory pathways, the recruitment of chemoreceptors to the different Che systems depends on protein specificity and the physical location of the Che modules [10], [11]. Structural studies have shown that receptor clusters are formed by interconnected heterotrimers of homodimers, which are associated with two CheWs and a dimer of CheA. Receptor homodimers can in turn form heterotrimers if they share common structural features and belong to the same class [12], [13]. The spatial segregation of MCPs to distinct cellular compartments also plays a role in the partitioning of MCPs among multiple CSS. For example, in *Rhodobacter sphaeroides*, membrane-associated and soluble MCPs are partitioned between polar and cytoplasmic clusters [10], [14].

We have been studying the multiple CSS of the Gram negative δ-proteobacterium *Myxococcus xanthus*. *M. xanthus* carries up to eight predicted chemosensory systems with 21 chemoreceptors [15], [16]. We speculate that the large number of CSS reflects the complexity of the *M. xanthus* life cycle, in which cells swarm as large groups to prey on other micro-organisms or build multicellular fruiting bodies [17]–[20]. Movement on surfaces does not employ flagella but instead requires two distinct motility machineries: polar retractile Type IV pili required for social (S) motility [21], [22] and distributed Agl-Glt complexes that form periodic foci and generate thrust for adventurous (A) motility [23]–[27].

Evidence suggests that *M. xanthus* motility behaviors are controlled by CSS. The Frz pathway, the first characterized Che-like system from *M. xanthus*, controls both motility systems by triggering periodic cellular reversals. This allows the bacteria to periodically reorient themselves, and may be similar to periodic switches in flagellar rotation, which allow the enteric bacteria to move along a chemotactic gradient by following a biased random swim. Motility is also regulated by Dif (Che2), a second sensory system that controls the production of surface exopolysaccharides in response to pilus activity [28]. Che4, a third CSS, also appears to be involved in the regulation of motility, although the specific mechanism remains unclear [29]. However, CSS are not exclusively dedicated to motility regulation in *M. xanthus*. In fact, the Che3 system regulates gene expression during fruiting body development [6], [29]–[32]. While future *M. xanthus* research on the exact contribution of each Che-like system to its life cycle will yield considerable biological insights, this task is complicated by the occurrence of 21 MCPs encoded in its chromosome, 13 of which are orphans. Furthermore, the activity of each CSS might be modulated by multiple MCPs as shown in other bacterial species. Cross-regulation and redundancies between additional pathways may also occur and thus further complicate the picture.

In this work, we set out to characterize each *M. xanthus* CSS and MCP and combine phylogenetic and cell biology analyses to examine their organization within cells to constitute functional modules. With this approach, we were able to show that MCPs belonging to the same phylogenetic group colocalize in cells and interact *in vivo* with components of CSS of their respective phylogenetic group. Protein-protein interaction analyses also suggest that colocalizing CSS belonging to same phylogenetic group constitute a unique large sensory module. Such organization is likely required to regulate complex cell behaviors such as biofilm and fruiting body formation. This analysis provides a broad perspective as to the function and organization of complex multicomponent chemosensory systems within bacterial cells and could be applicable to bacterial systems with similarly complex regulatory networks.

## Results/Discussion

### Identification of *M. xanthus* chemosensory modules

**Figure 1:**
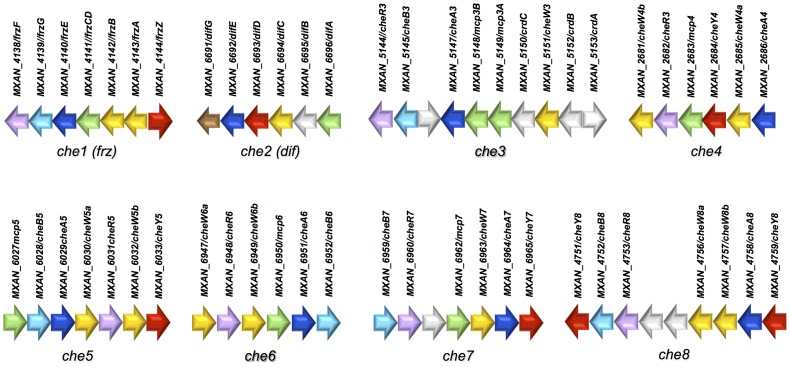
Genetic clusters carrying *che* genes in *M. xanthus*. Genetic organization of the genes composing the eight *che* clusters encoding the putative components of the chemosensory apparatus in *Myxococcus xanthus*. Predicted genes are indicated with their locus_tag, and their annotations and assigned names. The color code indicates homologous genes.

Four Che pathways have been characterized in *M. xanthus*: Frz, Dif, Che3 and Che4 [6], [29], [30], [33]. We used the conserved protein domain sequences from these pathways (Tables 1, 2 and 3) as queries to search for all *M. xanthus* Che homologues. Most *che* genes are organized in eight *che* operons, as previously described (Figure 1) [31]. Their predicted organization is depicted in Figure S1. The *M. xanthus* genome does not contain homologs of CheD and CheX or of CheZ, which are usually found in genomes of β- and γ-proteobacteria [1], [34]. None of the eight *che* clusters is located near known motility genes or other genes encoding cellular functions known to be controlled by CSS [6], [35], [13], [36].

**Table 1:** List of *M. xanthus* MCPs.^b^

Protein name	Locus tag (MXAN_)	Refseq	Length (aa)	Che operon	Functional domains (Pfam)				Transmembrane domain (TMHMM)		
					HAMP^a^ domain (position)	MCP^b^ signal domain (position)	Others domains (position)				
FrzCD	4141	ABF87130	417	*Frz*	-	(195 to 417)		-		-	
Mcp3B	5148	ABF90272	605	*che3*	(246 to 315)	(376 to 596)		-		6	
Mcp3A	5149	ABF91174	595	*che3*	(240 to 309)	(377 to 590)		-		6	
Mcp4	2683	ABF86464	538	*che4*	(189 to 258)	(322 to 538)		-		2	
Mcp5	6027	ABF86588	531	*che5*	(180 to 250)	(313 to 531)		-		2	
Mcp6	6950	ABF87794	547	*che6*	(184 to 253)	(335 to 546)		CHASE3^c^ (39 to 172)		2	
Mcp7	6962	ABF86943	821	*che7*	-	(317 to 534)		-		-	
DifA	6696	ABF91646	413	*dif*	(33 to 103)	(186 to 411)		-		2	
McpA	174	ABF91199	530	-	(180 to 250)	(318 to 530)		-		2	
McpB	227	ABF88952	553	-	(202 to 271)	(340 to 552)		CACHE^d^ (45 to 139)		1	
McpC	878	ABF90878	631	-	(280 to 351)	(416 to 631)		-		2	
McpD	1248	ABF89240	599	-	-	(386 to 598)		-		5	
McpE	1414	ABF92510	609	-	(259 to 329)	(392 to 609)		-		2	
McpF	2244	ABF92314	729	-	-	(502 to 720)		7TM diverse intracellular signalling		7	
McpG	2735	ABF92828	523	-	(170 to 240)	(299 to 521)		-		2 or 1	
McpH	3668	ABF87603	510	-	(160 to 230)	(293 to 510)		-		2	
McpI	3754	ABF87204	519	-	-	(184 to 497)		-		3	
McpJ	5907	ABF91268	723	-	(203 to 275)	(431 to 640) (636 to 723)		-		2	
McpK	6456	ABF92748	1048	-	-	(246 to 463)		Bacterial extracellular solute-binding proteins domain		3	
McpL	6938	ABF90672	591	-	-	(380 to 591)		-		5	
McpM	3453	ABF92699	737	-	-	(503 to 722)		7TM diverse intracellular signalling		7	

a =  Histidine kinase, Adenylate cyclase, Mcp, Phosphatase

b =  Methyl accepting Chemotaxis Protein

c =  Cyclase/Histidine kinases Associated Sensory Extracellular

d =  CAlcium channels and CHEmotaxis receptors

**Table 2:** List of *M. xanthus* CheA, CheW and CheY.

Protein name		Locus tag (MXAN_)	Refseq	Length (aa)	Che operon	Functional domains (Pfam)					
						Histidine-containing phosphotransfer (HPt) domain	Response regulator binding domain	Signal transducing histidine kinase, homodimeric domain	Histidine kinase domain	CheW-like domain	Response regulator receiver domain
**CheA**	FrzE	4140	ABF89991	777	*frz*	(9 to 108)	-	-	(365 to 508)	(513 to 644)	(661 to 774)
	DifE	6692	ABF92648	857	*dif*	(7 to 107)	(366 to 450)	(474 to 537)	(582 to 724)	(729 to 857)	-
	CheA3	5147	ABF90293	798	*che3*	(6 to 99)	-	-	(376 to 522)	(527 to 658)	(681 to 794)
	CheA4	2686	ABF90689	848	*che4*	(8 to 106)	-	-	(433 to 575)	(580 to 714)	(729 to 842)
	CheA5	6029	ABF88344	714	*che5*	(9 to 114)	-	-	(303 to 445)	(450 to 581)	(591 to 704)
	CheA6	6951	ABF88527	726	*che6*	(9 to 112)	-	-	(316 to 455)	(460 to 590)	(610 to 723)
	CheA7	6964	ABF92123	683	*che7*	(5 to 110)	-	(279 to 344)	(385 to 525)	(530 to 663)	-
	CheA8	4758	ABF85910	862	*che8*	(6 to 110)	-	(463 to 534)	(579 to 721)	(726 to 855)	-
**CheW**	FrzA	4143	ABF93093	160	*frz*	*-*	*-*	*-*	*-*	(16 to 154)	*-*
	FrzB	4142	ABF91972	112	*frz*	*-*	*-*	*-*	*-*	(26 to 109)	*-*
	DifC	6694	ABF92146	140	*dif*	*-*	*-*	*-*	*-*	(2 to 133)	*-*
	CheW3	5151	ABF89050	145	*che3*	*-*	*-*	*-*	*-*	(12 to 142)	*-*
	CheW4a	2685	ABF89623	194	*che4*	*-*	*-*	*-*	*-*	(59 to 186)	*-*
	CheW4b	2681	ABF90781	188	*che4*	*-*	*-*	*-*	*-*	(48 to 186)	*-*
	CheW5a	6030	ABF88929	182	*che5*	*-*	*-*	*-*	*-*	(42 to 174)	*-*
	CheW5b	6032	ABF86894	288	*che5*	*-*	*-*	*-*	*-*	(131 to 270)	(6 to 115)
	CheW6a	6947	ABF88743	159	*che6*	*-*	*-*	*-*	*-*	(7 to 142)	*-*
	CheW6b	6949	ABF87567	200	*che6*	*-*	*-*	*-*	*-*	(57 to 189)	*-*
	CheW7	6963	ABF85986	148	*che7*	*-*	*-*	*-*	*-*	(4 to 141)	*-*
	CheW8a	4756	ABF88564	174	*che8*	*-*	*-*	*-*	*-*	(28 to 165)	*-*
	CheW8b	4757	ABF90229	188	*che8*	*-*	*-*	*-*	*-*	(43 to 187)	*-*
	CheWa	4462	ABF92129	271	-	*-*	*-*	*-*	*-*	(10 to 130) (151 to 265)	*-*
**CheY**	FrzZ	4144	ABF89606	290	*frz*	-	-	-	-	-	(4 to 112)(170 to 282)
	DifD	6693	ABF87390	122	*dif*	-	-	-	-	-	(4 to 116)
	CheY4	2684	ABF88007	127	*che4*	-	-	-	-	-	(5 to 117)
	CheY5	6033	ABF92419	128	*che5*	-	-	-	-	-	(8 to 122)
	CheY7	6965	ABF88516	125	*che7*	-	-	-	-	-	(4 to 117)
	CheY8a	4751	ABF88585	126	*che8*	-	-	-	-	-	(7 to 120)
	CheY8b	4759	ABF90183	124	*che8*	-	-	-	-	-	(5 to 118)

**Table 3:** List of *M. xanthus* CheB and CheR.

Protein name		Locus tag (MXAN_)	Refseq	Length (aa)	Che operon	Functional domains (Pfam)			
						Response regulator receiver domain	CheB methylesterase	CheR methyltransferase, SAM binding domain	Tetratricopeptide repeat
**CheB**	FrzG	4139	ABF92096	334	*frz*	-	(150 to 330)	-	-
	CheB3	5145	ABF91845	352	*che3*	(7 to 118)	(162 to 342)	-	-
	CheB5	6028	ABF91242	355	*che5*	(9 to 122)	(160 to 338)	-	-
	CheB6	6952	ABF89213	353	*che6*	(8 to 115)	(166 to 346)		-
	CheB7	6959	ABF87336	348	*che7*	(9 to 121)	(163 to 346)	-	-
	CheB8	4752	ABF87312	345	*che8*	(9 to 120)	(157 to 340)	-	-
	CheBa	714	ABF90117	185	-	-	(6 to 164)	-	-
**CheR**	FrzF	4138	ABF86899	593	*frz*	-	-	(1 to 61) (74 to 270) **	(427 to 491) (496 to 556)
	CheR3	5144	ABF87122	440	*che3*	-	-	(69 to 262)	(358 to 406)
	CheR4	2682	ABF89636	485	*che4*	-	-	(74 to 256)	(357 to 419)
	CheR5	6031	ABF88773	413	*che5*	-	-	(72 to 250)	-
	CheR6	6948	ABF86317	569	*che6*	-	-	(73 to 255)	(467 to 535)
	CheR7	6960	ABF89444	273	*che7*	-	-	(77 to 267)	-
	CheR8	4753	ABF90193	290	*che8*			(82 to 278)	-
	CheRa	7103	ABF86029	346	-	-	-	(144 to 334)	-
	CheRb	713	ABF91568	278	-	-	-	(13 to 68) (81 to 270)**	-
	CheRc	2243	ABF89894	242	-	-	-	(47 to 141)*	-
	CheRd	2245	ABF91524	401	-	-	-	(72 to 182)*	-

* incomplete CheR domain.

** CheR short Nter domain + CheR S-adenosyl-L-methionine binding domain.

In addition to *che* operon encoded proteins, we identified several orphan *che* genes and 13 *mcp* genes dispersed throughout the chromosome (Tables 1–3). The other *M. xanthus* Che proteins with their respective locus tags, protein lengths and specific domains are listed in Tables 2 and 3. We did not conduct a thorough analysis of CheY homologs as the *M. xanthus* genome encodes 260 predicted response regulator domains (data not shown). In addition, it is impossible to distinguish if these proteins retain CheY function based on the sequence alone [1]. We reasonably assume that the response regulator domains encoded within the eight *che* operons constitute the minimum set of *M. xanthus* CheYs (Table 2).

### Deletions of *cheA* and *mcp* genes affect motility and fruiting body formation

**Figure 2:**
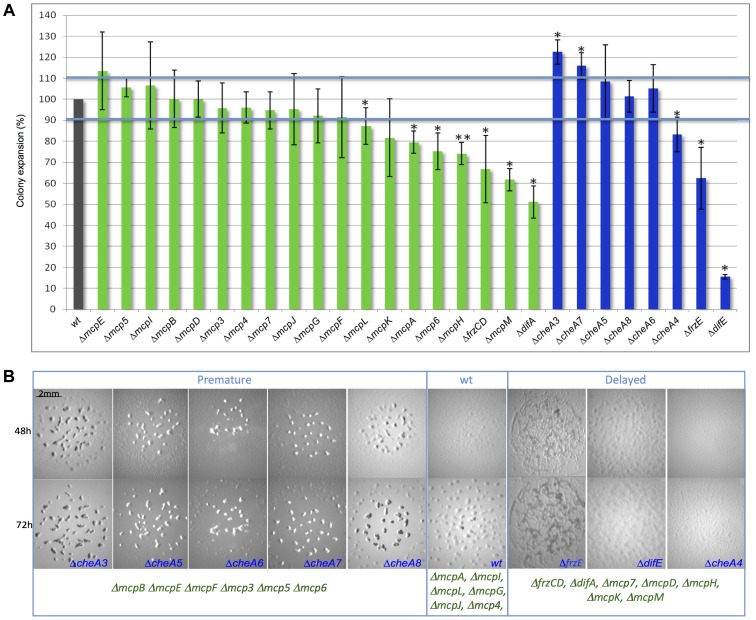
Motility and fruiting body formation defects of *Δmcp* and *ΔcheA* mutants. (A) Motility was measured after 48 h. Colony spreading of each mutant was normalized with that of a *ΔpilA* strain [72] completely incapable of S motility, to exclude cell growth effects. Error bars indicate standard deviations. One star corresponds to *p*<0.05; two stars correspond to *p*<0.005. (B) *ΔcheA* fruiting body formation images at 48 h and 72 h are shown. We identified classes of mutants developing earlier or later than wild type. Pictures of *Δmcp* fruiting bodies are shown in Figure S2. The blue color indicates *ΔcheA* mutants, green *Δmcp*.

In order to determine the function of the different MCPs and CSS during vegetative and developmental behaviors, we constructed a set of in-frame deletion strains in which all of the *mcp* and *cheA* genes were systematically deleted, with the exception of those for which an in-frame deletion in the wild-type strain DZ2 already existed (*frzCD*, *frzE*, *mcp3A*, *mcp3B* and *mcp4*) [6], [29], [37]. Deletions in *cheA3*, *cheA7*, *cheA4*, *mcp6*, *mcpA*, *mcpH*, *mcpL* and *mcpM* caused S-motility defects, which significantly reduced or enhanced colony spreading compared to wild-type (*p*<0.05) (Figure 2A). This was also true for *ΔdifE*, *ΔfrzE*, *ΔdifA* and *ΔfrzCD*, for which a S-motility defect has already been described [33], [38].

At least 13 *Δmcp* and all *ΔcheA* strains were defective in fruiting body formation, showing altered developmental timing or displayed a complete absence of development (Figures 2B and S2). *M. xanthus* fruiting body formation requires a functional motility apparatus. Therefore, in *ΔcheA4*, *ΔcheA7*, *ΔmcpH*, *ΔmcpM* and *Δmcp6* strains, the developmental defects might result from the motility defects also shown by these mutants (Figure 2A and B). However, in most cases the two phenotypes are unrelated, suggesting that most Che proteins either regulate motility exclusively during development or are involved in functions other than motility in *M. xanthus*. In order to check whether the *Δmcp* and *ΔcheA* strains were capable of A motility, we systematically deleted the *pilA* gene in each *Δmcp* and *ΔcheA* strain to exclude an effect of S motility, as this motility system is active on the substrate commonly used to test A motility (1.5% agar plates) [37]. All double mutants displayed individual cells at the colony edges suggesting the presence of a functional A-motility system (Figure S3). Notably, we were unsuccessful at deleting *mcpC*, suggesting that this gene might be essential in *M. xanthus*. In our assays, mutants lacking McpG, McpI, McpJ, McpL and Mcp4 did not display any defects (Figures 2 and S2). Among these MCPs, McpI and McpL are not expressed in cells (see below), similar to that observed in the *R. sphaeroides cheOp1* operon [39]. In the case of Mcp4, McpG and McpJ, which were clearly expressed in cells (see below), the corresponding mutants might display insignificant defects or have functions masked by the presence of another MCP.

Interestingly, most *cheA* deletions caused more severe defects than deletions of *mcp* genes from the same operons. These results support the hypothesis that each CSS is activated by multiple receptors, as CheAs are core components of CSS. Thus, phenotypic analyses can be ambiguous for the purpose of clustering *M. xanthus* MCPs into functional modules. Indeed, MCPs showing opposing functions may still signal to the same Che pathway and contribute differently to the final response. For example, it has been recently shown that the Tar and Tsr *E. coli* chemoreceptors, both signaling to the same CheA, show opposite pH-taxis responses [40].

### *M. xanthus* MCP and Che proteins show similar phylogenetic distributions

**Figure 3:**
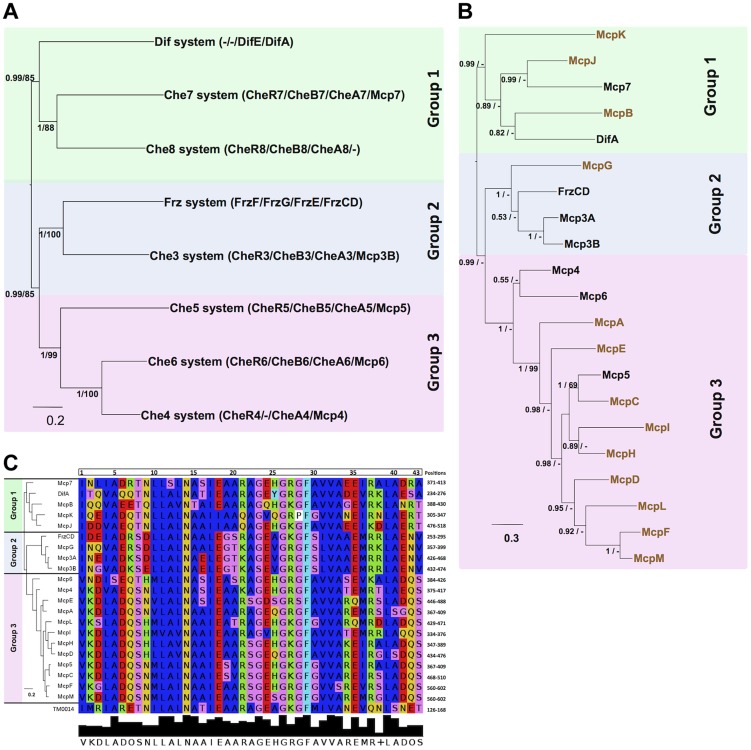
*M. xanthus* MCPs and CSS are organized in three taxonomic groups. (A) Concatamers of *M. xanthus* Che protein sequences were generated as described in Methods. Based on PP values, the eight concatamers can be divided into Group 1 (green background), Group 2 (blue background) and Group 3 (pink background). (B) The tree generated for the 21 *M. xanthus* MCP homologs shows a similar partition in three groups. The MCPs in black belong to *che* operons, while the MCPs in color are the orphans. (C) A tree generated with the MCP conserved protein sequences involved in the MCP-CheW interaction (Vu et al., 2012) gives rise to the same distribution as in (B). The alignment of the protein sequences involved in the MCP-CheW interaction from *T. maritime* [41] and *M. xanthus* MCPs is shown. Colors indicate residues with the same properties. Numbers at nodes in (A) and (B) indicate posterior probabilities (PP) computed by MrBayes and bootstrap values (BV) computed by PhyML. Only PP and BV above 0.5 and 50% are shown. The scale bars represent the average number of substitutions per site.

To obtain additional insights on MCP-CSS associations in *M. xanthus*, we compared the phylogeny of the MCPs to the phylogeny of the CSS, reasoning that MCPs and CSS that share the same phylogenetic distribution might be functionally associated. We started by determining the phylogenetic associations among the eight *M. xanthus* Che clusters. First, we obtained the individual phylogenies of the MCP, CheA, CheW, CheR and CheB proteins from those clusters. The five individual phylogenies showed similar topologies (Figure S4). However, as these phylogenies were based on a limited number of unambiguously aligned positions and the nodes of the inferred trees were often weekly supported (PP<0.5), we concatenated the MCP, CheA, CheR and CheB sequences from each locus into a super-sequence and used the resulting supermatrix to obtain phylogenetic trees with a higher resolution (Figure 3A). Whenever a Che cluster contained two homologues of uncertain orthologous relationship, we excluded them from the concatenation. This was the case for CheY-like and CheW-like proteins (Table 2). In the case of the Che3 system, Mcp3A and Mcp3B derive from a recent duplication in the Cystobacterineae (unpublished) and thus Mcp3B was included in the supermatrix. The tree obtained from the concatenated data sets was significantly more resolved than the individual trees (Figure 3A and Figure S4). Figure 3A shows that the Che clusters may be categorized in three main groups: Group 1 containing Dif, Che7 and Che8; Group 2, FrzCD and Che3; Group 3, Che4, Che5 and Che6.

Next, in order to assign the 13 orphan MCPs of *M. xanthus* to a Che system, we performed a phylogenetic analysis of the 21 MCPs. The resulting MCP tree was strongly correlated. Specifically, the 21 MCPs formed three major monophyletic groups (PP = 0.99, Figure 3B) with the first group containing five MCPs (McpB, McpJ, McpK, DifA and Mcp7). Phylogenetic analyses suggest that Mcp7 and McpJ emerged upon a recent gene duplication event and that McpB is closely related to DifA (data not shown). Group 2 contains FrzCD, McpG, Mcp3A and Mcp3B and Group 3, the largest group, contains all of the remaining MCPs. In Groups 2 and 3, the MCPs are strongly associated and therefore may have emerged by recent gene duplication events in the δ-proteobacteria.

The congruence between the MCP and CSS distributions suggests that phylogenetic relationships may be useful in predicting MCP-Che associations. These associations should be reflected in binding specificities such that MCPs that interact with the same downstream Che module should have similar CheW-binding motifs. It has been recently shown that a short peptide sequence is involved in MCP-CheW binding in *T. maritima* [41], [42]. All *M. xanthus* MCPs contained a conserved predicted CheW-binding motif (Figure 3C). Such motifs were aligned and the alignment was used to construct a phylogenetic tree. Although the nodes were poorly supported due to the short sequences, the resulting tree presented the same topology observed in Figure 3B. This analysis further suggests that MCPs belonging to the same group have similar binding specificities and are associated with the same CheW and, therefore, Che system.

The MCP C-terminal methylated domain is constituted by a repetition of several heptamers. MCPs can be classified depending on the number of these heptamers and, therefore, on the length of the C-terminal region [12]. It appears that MCPs of different lengths cannot form trimers of dimers as has been described for the *P. aeruginosa* McpB and WspA belonging to class 36H and 40H [43], [44], or *R. sphaeroides* McpG and TlpT belonging to classes 34H and 36H [45], [46]. Sequence analyses based on the Alexander and Zhulin classification show that all *M. xanthus* MCPs belong to class 40H, with the exception of DifA and McpK which belong to class 44H [15]. This result suggests that DifA and McpK interact with each other and signal to the Dif system forming a separate module.

Taken together, the phylogenetic and sequence analyses suggest the following associations: DifA and McpK linked to Dif; Mcp7, McpB and McpJ linked to Che7 and Che8; FrzCD, McpG, Mcp3a/Mcp3B linked to Frz and Che3; all remaining Mcps linked to Che4, Che5 and Che6.

### Subcellular localization of *M. xanthus* MCPs

**Figure 4:**
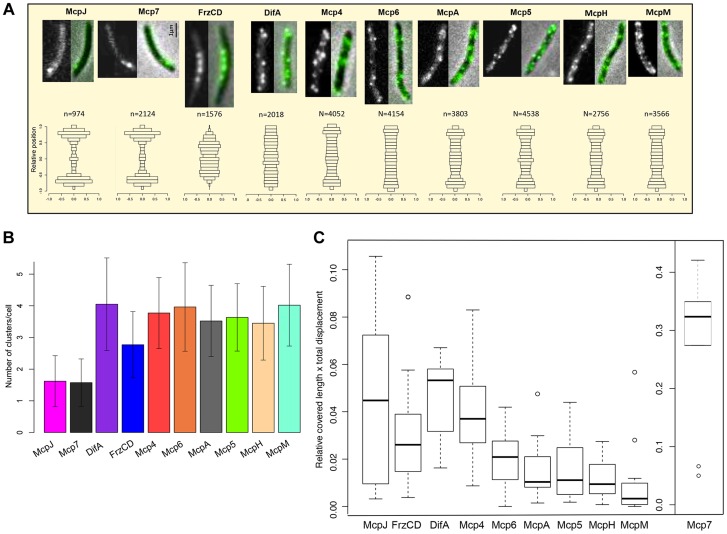
MCP-GFP fusions localize in multiple dynamic clusters in cells. (A) In the first row, fluorescence (left) and overlay between fluorescence and phase contrast images (right) are shown for each MCP-GFP. In the bottom row, *n* clusters (numbers indicated above the histograms) were analyzed for each *mcp-gfp* strain and their relative position in cells in the *y*-axis is shown (0.0 indicate the center of the cell along the *y*-axis). Bars indicate the fraction of clusters localizing in the corresponding position in the *y*-axis. (B) Average number of clusters for each MCP-GFP. (**C**) Box plots indicate the medians of the product of the relative cell length and the total distance covered by the MCP-GFP clusters *  = *p*<0.05; **  = *p*<0. 5E-04 (refer also to Methods, Table S2 and Figure S4).

The *R. sphaeroides* chemosensory network is composed of two sensory modules each including multiple receptors [13], [31], [47], [48]. The two modules are physically separated in cells, as one constitutes a transmembrane polar cluster and the other one a cytoplasmic cluster [11], [46], [49]. We hypothesized that in *M. xanthus*, much like in *R. sphaeroides*, MCPs belonging to the same sensory module should have similar localization patterns [45], [46]. To test this hypothesis, we constructed strains that expressed the C terminus of each MCP fused to the green fluorescent protein (eGFP). Each gene fusion was placed at the respective endogenous locus and were shown not to interfere with cellular functions, with the exception of FrzCD-GFP and DifA-GFP which only partially complemented the motility defects observed in the respective deletion mutants (Figure S5) [50]. The strains were then examined *in vivo* by live-fluorescence microscopy. Ten of the 21 MCP-GFP fusions were highly expressed in cells, showing bright fluorescent foci and clear localization. Conversely, the remaining fusions showed only weak and diffused fluorescent signal in vegetative conditions (Figures 4A, S6 and S7). Mcp3B, McpE and McpG that we could not detect by fluorescence microscopy during vegetative growth, were instead expressed during development (Figure S7). MCPs that we could not detect in any condition, but that clearly play a role during development, were probably expressed at low levels, which is also the case with the very low-abundance receptors Trg and Tap of *E. coli* [51], [52].

We proceeded with the analysis of the ten fusions that were localized in all conditions: DifA, FrzCD, Mcp7, McpJ, Mcp4, Mcp5, Mcp6, McpA, McpH and McpM. These MCP-GFP strains all showed multiple fluorescent clusters at the cell poles, the cell periphery or the cytosol (Figure 4A and B). To analyze these patterns we designed a procedure allowing large-scale automated image acquisition and analysis of the clusters formed by each MCP-GFP fusion (refer to Methods). With this approach, we obtained a localization map of each MCP by assigning the detected clusters to their relative cellular position and identified three main localization patterns. Mcp7 and McpJ formed only one or two clusters at the subpolar cell regions (Figure 4A and 4B). FrzCD showed a unique localization pattern with cytoplasmic foci excluded from the poles and occupying the central region of the cell body, as previously described [50], [53]. The remaining MCPs (DifA, Mcp4, Mcp5, Mcp6, McpA, McpH, McpM) formed foci distributed all along the periphery of cells, as predicted by the presence of transmembrane domains in their sequence (Table 1, Figure 4A and 4B). As all of the MCP foci appeared to be dynamic, we systematically analyzed the dynamics of these foci in single cells (Movie S1–S3, Figure S8). In order to exclude any interference from cellular movements, MCP foci were tracked in non-motile cells. Our analyses revealed that Mcp7 was significantly more mobile than all the other MCPs (*p*<0.005) (Figure 4C, Figure S8C and Table S2). Also, DifA and Mcp4 clusters were significantly more mobile than Mcp5, Mcp6, McpA and McpH fusions which were more static while McpM which showed little mobility if any (Figure 4C, S8B, S8C and Table S2). Interestingly, while the more static McpM carries the highest number of transmembrane domains, the faster Mcp7 is a cytoplasmic protein (Table 1). However, while FrzCD also lacks transmembrane domains, it shows slower movement rates compared to Mcp7. This might be explained by the anchoring of the FrzCD clusters to some intracellular structures [53], [54].

Based on localization and phylogeny, we can postulate that (i) McpJ is linked to Mcp7, (ii) FrzCD constitutes a sensory module by itself and (iii) MCPs of Group 3 are linked to the Che4, Che5 or Che6 pathways (Figures 3 and 4). Although the localization and dynamics of DifA suggest that it interacts with the receptors of Group 3, this appears unlikely from the divergence of their respective C-terminal domains based on phylogenetic and sequence analyses (see above) [12]. This cellular localization and dynamics of the chemoreceptors is largely consistent with the functional groups suggested by the phylogenomic analysis described above.

**Figure 5:**
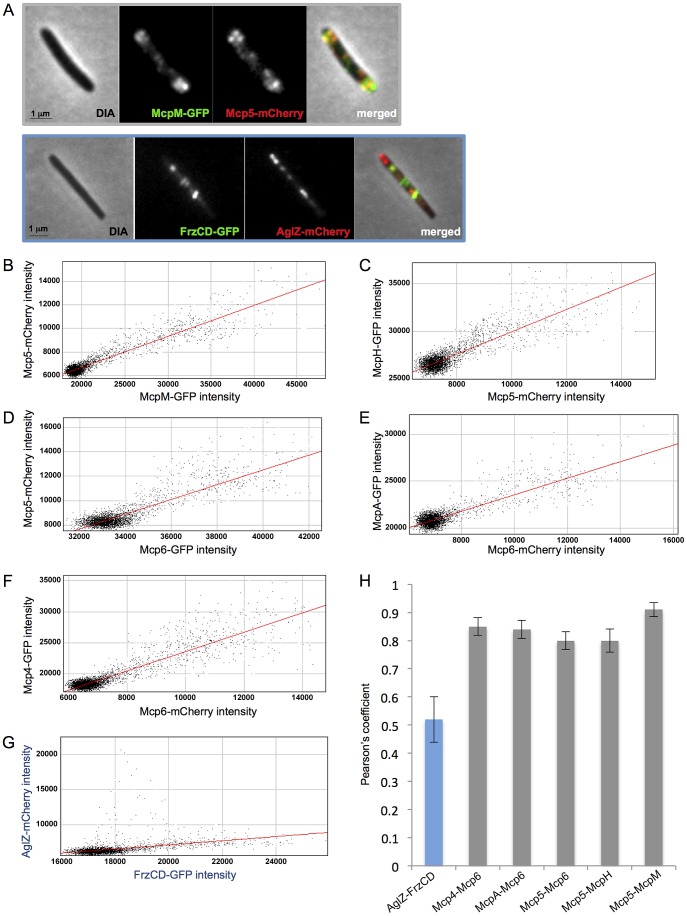
MCPs colocalization analysis. (A) Fluorescence micrographs of *mcp5-mCherry mcpM-gfp* and *frzCD-gfp aglZ-mCherry* cells are shown as examples. From (B) to (G) scatterplots of individual red and green pixel intensities of double-labeled cells are shown. (H) Average Pearson's correlation coefficients (PCCs) each calculated from ten scatterplots per strain.

To verify that Mcp4, Mcp5, Mcp6, McpH, McpA and McpM, predicted to be associated in the same functional module, are colocalized in cells, we constructed *M. xanthus* strains expressing two fluorescently labeled MCPs, with either the GFP or the mCherry. Fluorescence micrographs of each strain were taken and colocalizations were quantified for 20 cells per double-labelled strain. Quantifications were determined by calculating the Pearson's coefficient that measures the degree of linear dependence between the localization of a red signal and the localization of a green signal in the same cell [55]. Our analyses showed that Mcp5-Mcp6, Mcp5-McpH, Mcp5-McpM, Mcp6-Mcp4 and Mcp6-McpA significantly colocalized in cells (Pearson's coefficient >0.7) (Figure 5). We used *frzCD-gfp/aglZ-mCherry* cells as negative control because FrzCD and AglZ have previously been shown to be exclusively localized in cells (Figure 5) [53].

### Che4, Che5, Che6 and multiple MCPs might constitute a large chemosensory module

**Figure 6:**
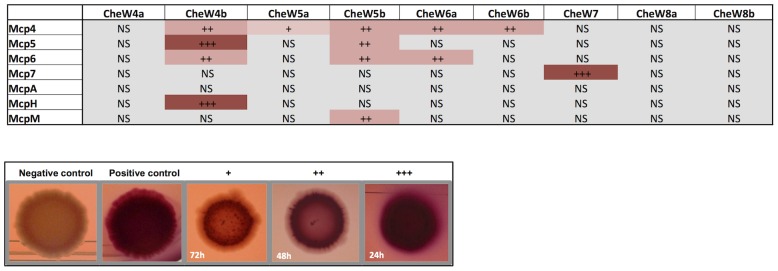
*In vivo* MCP-CheW interactions. Bacterial two-hydrid assays on plates. Interactions between MCPs and CheWs are shown. +++, ++ and + indicate bacterial colonies turning red within 24 h, 48 h and 72 h respectively. “NS” (not significant) means that the colony color was as the negative control. We only show interactions resulting positive for both the pUT18C*mcp*/pKT25*cheW* and pKT25*mcp*/pUT18C*cheW* combinations and reproducible in two experiments performed in triplicate. Examples of colonies from negative control (empty plasmids); positive control (pUT18C*mcp7*/pKT25*cheW7*); + (pUT18C*mcpM*/pKT25*cheW4b*); ++ (pUT18C*mcp4*/pKT25*cheW4b*); +++ (pKT25C*mcpM*/pUT18C*cheW4b*) are shown.

The phylogenetic and localization studies suggest that a large number of MCPs are recruited by the Che4, Che5 and Che6 pathways. To directly assess this hypothesis, we tested the interactions between Mcp4, Mcp5, Mcp6, McpH, McpA and McpM with all CheW-like proteins from the Che4, Che5 and Che6 pathways in a bacterial two-hybrid assay. Since these chemoreceptors were not predicted to interact with the Che7 and Che8 pathways, we included CheW homologs from these pathways as specificity controls. The interaction between Mcp7 and CheW7 was also used as a positive control. Except for McpA for which no interaction was detected with any of the tested CheW homologs, all tested MCPs interacted with at least one CheW from Che4, Che5 or Che6 (Figure 6). Remarkably a high level of specificity was observed in some cases: for example, McpM only interacted with CheW5b and McpH only interacted with CheW4b. In other cases, one MCP could interact with several CheW proteins: specifically, Mcp4 interacted with all of the CheWs except for CheW4a and the negative controls; Mcp5 interacted with CheW4b and CheW5b; and Mcp6 interacted with CheW5b and CheW6a. As expected, none of these receptors interacted with CheW7, which specifically interacted with Mcp7, nor with CheW8a and CheW8b, which are phylogenetically distant (Figure 6). Together, these results raise the possibility that *M. xanthus* Che utilizes higher order chemosensory modules comprised by several MCPs and Che pathways. The two-hybrid analysis suggests that each CheW has binding specificities that can be used to recruit multiple specific MCPs to a given signaling complex.

**Figure 7:**
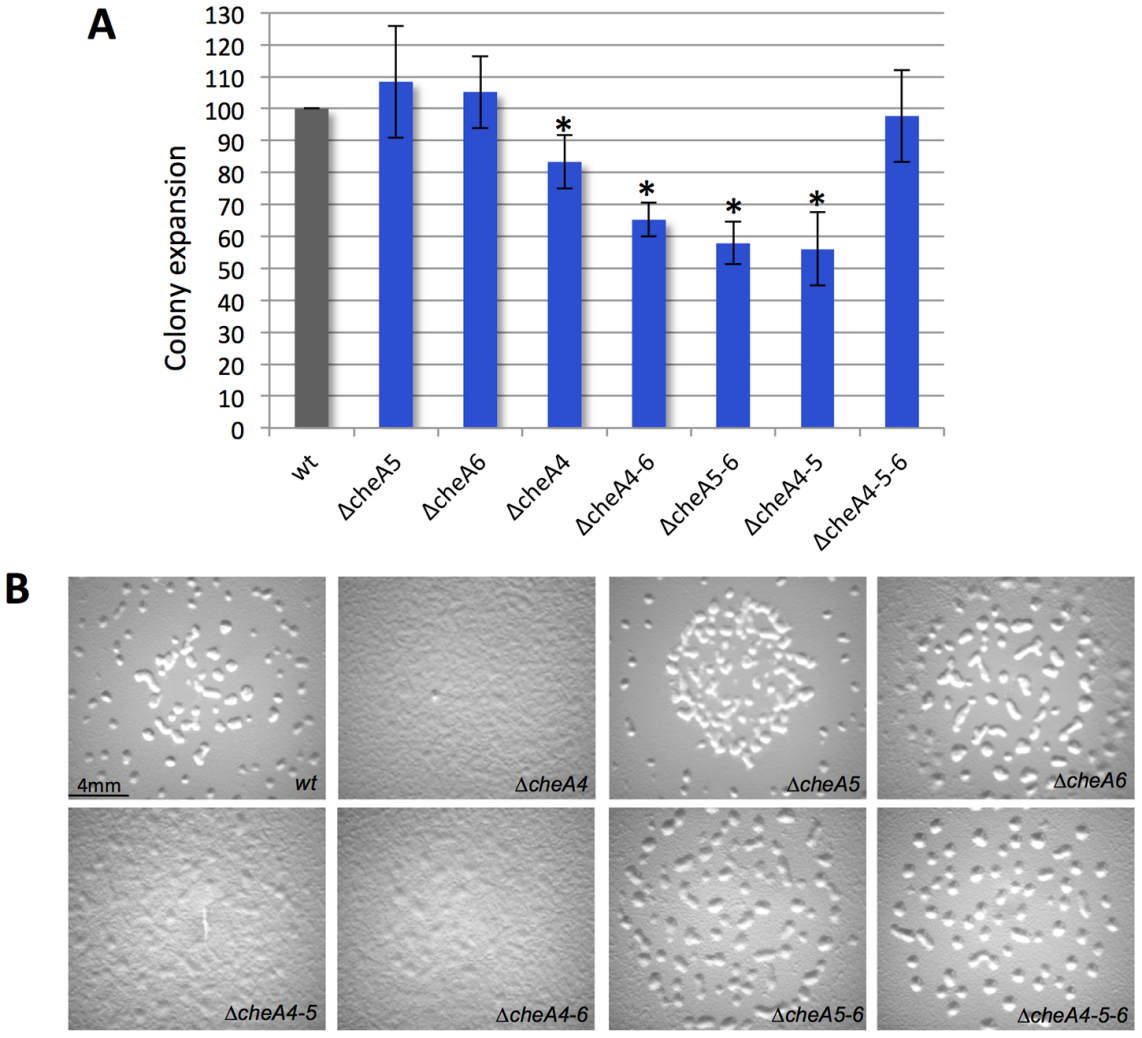
*ΔcheA* triple mutants have restored phenotypes as compared to single and double mutants. (A) Motility was measured after 48 h. The colony spreading of each mutant was normalized with the one of a *ΔpilA* strain [68] completely incapable of S motility, to exclude cell growth effects. Error bars indicate standard deviations. The star corresponds to *p*<0.005. (B) *ΔcheA* fruiting body formation images at 72 h are shown.

To further test the existence of a module comprised by the Che4, Che5 and Che6 systems and receptors, we combined deletions of *cheA4*, *cheA5* and *cheA6* and analyzed motility and developmental phenotypes. Interestingly, *ΔcheA4*, *ΔcheA5* and *ΔcheA6* double mutants are significantly more affected in S motility and fruiting body formation than single mutants (Figure 7). However, these phenotypes are restored to wild type in a *ΔcheA4ΔcheA5ΔcheA6* triple mutant. While this analysis does not reveal the precise biological function of the Che4, 5 and 6 pathways, it shows that the lack of two CheAs from this module deregulates the remaining CheA. This result strongly suggests that CheA4, CheA5 and CheA6 are part of the same regulatory module.

### Conclusions

In this study we sought to understand the partitioning of *M. xanthus* chemoreceptors among eight CSS to constitute sensory modules. We hypothesized that Che modules might attract multiple receptors and Che proteins as observed in other bacterial species and that the analysis of their cellular organization would help us to understand the role of these proteins in the *M. xanthus* life cycle. For this purpose, we first compiled a full list of the putative *M. xanthus* Che proteins and chemoreceptors. We were not surprised to find a total of 67 proteins as, in most cases, the number of one- and two-component systems present in a bacterial genome directly relates to the complexity of the life cycle [56]. The same might be true for CSS. Our systematic deletion of the 21 *M. xanthus* chemoreceptors and CheA encoding genes revealed that two thirds of them are involved in the temporal regulation of fruiting body formation, a multi-step differentiation process requiring the perception of numerous signals for the activation of key regulation check-points [20], [57].

**Figure 8:**
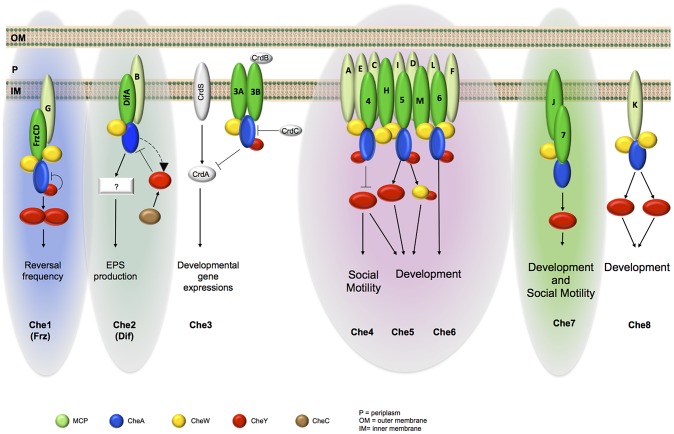
Schematic organization of *M. xanthus* Che modules as depicted from phylogenetic, cell biology and protein interaction analyses. For clarity, we omitted CheR and CheB proteins and do not specify the MCP-CheW interactions. MCPs in light green are the ones for which interactions with a CSS have not been demonstrated. The different color backgrounds indicate taxonomic Group 1 (green), Group 2 (blue) and Group 3 (pink). Group 1 was further divided in two subgroups labelled with light and dark green, based on the localization analysis.

Based on an integrated approach, we found that MCPs and CSS show comparable phylogenetic distributions in three main groups and that MCPs belonging to the same phylogenetic group colocalize. In particular, MCPs of Group 3 seemed to constitute a large chemosensory module together with three CSS, namely Che4, Che5 and Che6 (Figure 8). The presence of such a complex array of chemosensory proteins suggests that social behaviors such as cell group motility and biofilm formation might require interwoven regulatory systems composed by multiple Che-like systems and that the final cellular responses are generated following both the integration of signals transduced by different MCPs at the CheA level and the interaction among different Che systems. Also, cross-regulation between different Che systems can add an additional layer of complexity, as suggested by previous work showing inter-dependence between the Frz and the Dif pathway [58]. Once the composition of each module has been dissected, it will be possible to identify their signals and outputs to clarify their precise function in the *M. xanthus* life cycle.

It has recently been reported that multiple chemosensory systems occur as frequently as single ones, highlighting the importance of investigating model microbes that encode multiple chemosensory systems [1]. By providing a broad perspective on how a complex multicomponent chemosensory apparatus is arranged within cells, this work establishes a basis for a deeper analysis on how signals are perceived, integrated and translated in cell behaviors at the level of each chemosensory module. Analogous approaches could be applied to bacterial systems with similarly complex regulatory networks.

## Materials and Methods

### Bioinformatics analysis of *che* genes

Protein sequences were analyzed by Pfam (release 24.0) (see comment on Tables 1, 2 and 3) databases [59]. Signal peptides and transmembrane helices were predicted using the signalP 3.0 [60] and TMHMM v.2.0 [61] servers, respectively. Genomic regions were investigated using the complete genome sequence available on NCBI [15].

For the dataset construction and phylogenetic analyses, *M. xanthus* Che and MCP homologues were retrieved from the complete *M. xanthus* DK 1622 proteome available on NCBI (http://www.ncbi.nlm.nih.gov/genome/proteins/1120/?project_id=58003; [15] using Blastp with default parameters [62]. The distinction between homologous and non-homologous sequences was assessed by visual inspection of each Blastp outputs (no arbitrary cut-off on the E-value or score). To ensure the exhaustive sampling of homologues, iterative Blastp queries were performed using homologues of *M. xanthus* MCP identified at each step as new seeds.

The retrieved homologues were gathered in a dataset and the corresponding sequences were aligned using the ClustalW2 program (Default parameters, [63]. Each alignment was visually inspected and manually refined when necessary using the ED program from the MUST package [64]. Regions where the homology between amino acid positions was doubtful were manually removed using NET from the MUST package.

Both Maximum likelihood (ML) and Bayesian phylogenetic trees were computed for the MCPs. ML analyses were run using PhyML version 3.0 with the Le and Gascuel (LG) model (amino acid frequencies estimated from the dataset) and a gamma distribution (4 discrete categories of sites and an estimated alpha parameter) to take into account evolutionary rate variations across sites [65]. The robustness of each branch was estimated by the non-parametric bootstrap procedure implemented in PhyML (100 replicates of the original dataset with the same parameters). Bayesian analyses were performed using MrBayes [66] with a mixed model of amino acid substitution including a gamma distribution (4 discrete categories) and an estimated proportion of invariant sites. MrBayes was run with four chains for 1 million generations and trees were sampled every 100 generations. To construct the consensus tree, the first 1500 trees were discarded as “burnin”.

In order to obtain a high-resolution taxonomic distribution of the Che systems, we combined the CheR, CheB, CheA and MCP conserved sequences in a so-called supermatrix. When more than one homologue of these genes was present in a given genome, the genes were combined according to their physical linkage on the chromosome.

### Bacterial strains, plasmids and growth

Strains and plasmids are listed in Table S1. *M. xanthus* strains were grown at 32°C in CYE rich media as previously described [37]. Plasmids were introduced into *M. xanthus* cells by electroporation. Deletion and MCP-GFP fusions were inserted in frame to avoid polar effects on the downstream gene expression. These strains were obtained by homologous recombination based on a previously reported method using the pBJ113 or pBJ114 vectors [37]. The codon regions that we deleted to obtain *Δmcp* and *ΔcheA* in frame deletion strains are specified in Table S1. To generate strains expressing MCP-GFP fusion proteins, we constructed DNA cassettes including the last approximately 800 bp of each *mcp* gene, with the exception of the stop codon; the gene encoding the *egfp* gene from the pEGFP-N1 plasmid (Invitrogen) excluding the start codon and including the stop codon; the intergenic region between the *mcp* gene of interest and its immediately downstream gene, if any; the first 800 bp of the *mcp* downstream gene. Between the *mcp* gene fragment and the *egfp* we inserted the following linker: CGG GAT CCA CCG GTC GCC ACC.

To obtain *Δmcp/pilA::tet* and *ΔcheA/pilA::tet* strain, we systematically electroporated *Δmcp* and *ΔcheA* cells, as previously described, with genomic DNA from strain DK10407 [37].

*Escherichia coli* cells were grown under standard laboratory conditions in Luria-Bertani broth supplemented with antibiotics, if necessary.

For phenotypic assays, cells (5 µl) at a concentration of 5×10^9^ cfu ml^−1^, were spotted on CF-agar plates or CYE plates containing an agar concentration of 0.5 or 1.5%, incubated at 32°C and photographed after 24 h, 48 h, 72 h and 5 days with an Olympus SZ61 binocular stereoscope. To measure the fluorescence intensity of MCP-GFP fusions during developmental conditions, cells were grown in submerged cultures in CF medium for 48 h as previously described [67].

### Bacterial two-hybrid experiments

Bacterial two-hybrid experiments, plate were performed as previously described [68] and as recommended by the manufacturer instructions (Euromedex).

### Fluorescence microscopy

For fluorescence microscopy analysis, 5 µl of cells from 4×108 cfu ml^−1^ vegetative CYE or submerged CF cultures were spotted on a thin fresh TPM agar [69] pad atop a slide. A cover slip was added immediately on the top of the pad, and the obtained slide was analyzed by microscopy using a Nikon Eclipse TE2000 E PFS inverted epifluorescence microscope (100× oil objective NA 1.3 Phase Contrast) [70]. Typical time-lapse movies were shot for 20 min or 3 min with frames captured every 30 or 5 s, respectively. Movies were obtained by processing the series of images collected with Image J (Rasband, W.S., ImageJ, U. S. National Institutes of Health, Bethesda, Maryland, USA, http://imagej.nih.gov/ij/, 1997–2012.) and FIJI [71].

Alternatively, 1 µl of cells from 4×109 cfu ml^−1^ vegetative CYE cultures were spotted on pretreated 96-well Angiogenesis glass microplate (IBiDi). 50 µl of 37 C, 2% Low melting agarose (Sigma) were immediately placed on the top of the cell drop and the glass slides were left 30 minutes at room temperature before being imaged. The microscope screening of a complete microplate was obtained with a fully automatized system. The microscope devices were optimized in order to minimize the mechanical moves and provide rapid autofocus capability (epi/diascopic diode lightening, piezo-electric stage). The microscope and devices were driven by a recently released Nikon-NIS software called “JOBS”.

### Image analysis

Image analyses were performed with ImageJ or Fiji. Kimographs were obtained from 3 min time-lapse movies with frames captured every 5 s. From these movies, areas corresponding to selected non-moving cells were cropped. A line with the same thickness, length and curvature of a selected cell was manually drawn inside this cell. Cells were straightened with the function “reslice” to obtain the kymograph. From the kymographs, we measured the total distance (the total number of pixels covered horizontally) as well as the relative cell-body length covered by each cluster, regardless of the direction (red and blue, respectively, in Figure S8B). Then we considered the product between the total displacement and the relative covered length as a measurement of the degree of dynamism of each MCP.

The position of MCP-GFP clusters along the major axis of the cell was obtained with a workflow described in Figure S9 and automated with a Python script in FIJI. As the signal to noise ratio varied among different strains, it was necessary to adapt the analysis for each MCP-GFP in order to obtain a realistic distribution of clusters. The fluorescence intensity was calculated with FIJI by subtracting from the measured fluorescence intensity of each cell the background measured in the same frame. Colocalizations were calculated with the plugin JACoP from FIJI.

### Statistical analysis

Relative net swarming in Figure 2 was calculated as the average ratio between the surfaces in pixels of the swarming mutant versus wild-type colonies (100%) after 48 h. Surfaces were normalized with the surface of a *ΔpilA* mutant (0%). The averages were obtained from at least three independent experiments performed in duplicates. Student's T-tests were used to determine the statistical significance. The relative cell length and the total distance covered by MCP were determined on 20 clusters by calculating, with the software R, data medians and Interquartile Ranges (IQRs: defined as the difference between the third and the first quartiles of the data) respectively. Statistical significance was calculated by using Wilcoxon tests.

## Supporting Information

Figure S1 Schematic diagram of the putative organization of *M. xanthus* Che systems and orphan MCPs. Chemosensory proteins might form complexes analogously to their enteric counterparts.
(TIF)

Figure S2 Fruiting body formation phenotypes of *Δmcp* mutants. Cells (5 µl), at a concentration of 4×10^9^ cfu ml^−1^, were spotted on CF plates containing an agar concentration of 1.5%, incubated at 32°C and photographed after 24, 48 and 72 h with a Olympus SZ61 microscope. Pictures taken at 48 h are shown.
(TIF)

Figure S3 A-motility phenotypes of *Δmcp/ΔcheA, pilA::tet* double mutants. Cells (5 µl), at a concentration of 4×10^9^ cfu ml^−1^, were spotted on CYE plates containing an agar concentration of 1.5%, incubated at 32°C and the edge of each colony was photographed after 48 h with a 10x objective.
(TIF)

Figure S4 Phylogenetic relationships between *M. xanthus* Che homologues. Numbers at nodes indicate posterior probabilities (PP) computed by MrBayes and bootstrap values (BV) computed by PhyML. Only PP and BV above 0.5 and 50% are shown. The scale bars represent the average number of substitutions per site.
(TIF)

Figure S5 S motility and fruiting body formation phenotypes of a *difA-gfp* strain. Cells (5 µl), at a concentration of 4×10^9^ cfu ml^−1^, were spotted on CF-agar plates or CYE plates containing an agar concentration of 1.5% or 0.5%, respectively, incubated at 32°C and photographed after 48 or 72 h with a Olympus SZ61 microscope.
(TIF)

Figure S6 Mcp-GFP fusions are expressed in cells. Western blots using antibodies against GFP (Invitrogen) show stable expression of Mcp-GFP chimeras. Whole cell extracts were prepared from cells grown in liquid CYE media to mid-log phase. Ten micrograms of total proteins were loaded into 10% SDS-polyacrylammide gels. Proteins were then transferred to nitrocellulose membrane and immonoblots were prepared as previously described [50].
(TIF)

Figure S7 Cells expressing MCP-GFP proteins were grown in rich medium up to OD600 = 0.5 or incubated in CF medium in submerged cultures for 48 h as described by Kuner and Kaiser [67]. Cells were then collected and imaged at the fluorescence microscope as described in Material and Methods. The fluorescence intensity of approximately 200 cells per strain was measured with Fiji. We show (**A**) the fluorescence intensity from vegetative sample and (**B**) the ratio between the fluorescence intensity from developmental and vegetative samples. All values were normalized with the fluorescence intensity of wildtype samples. T tests were used to verify that the fluorescence intensity of the *mcp-gfp* strains was significant as compared to wt (*  = *p*<5E-04). Examples of fluorescence images are shown in (**C**).
(TIF)

Figure S8 Mcp clusters are dynamic. (**A**) Time-lapse fluorescence microscopy of single cells imaged every 30 seconds. (**B**) Kimographs obtained from time-lapse fluorescence microscopy of single cells imaged every 5 seconds. For the cytoplasmic FrzCD-GFP, kimographs were realized by analyzing cells from one pole (−1) to the other (0). For the remaining trasmembrane MCP-GFP fusions, kimographs were realized by analyzing the whole perimeter of cells from one pole (−1) to the other (0) and, then, returning to the first pole (+1). Red and black arrows indicate static and dynamic clusters, respectively. (**C**) Box plots indicate the medians of the distance covered in a given time by the MCP-GFP clusters. *  = *p*<5E-04 (See also Methods and Table S2).
(TIF)

Figure S9 Image J based work flow used to detect MCP-GFP clusters.
(TIF)

Table S1 Strains and plasmids.
(PDF)

Table S2 T values (Wilcoxon tests).
(PDF)

Movie S1 *M. xanthus* individual cell expressing Mcp7-GFP.
(AVI)

Movie S2 *M. xanthus* cells expressing a DifA-GFP.
(AVI)

Movie S3 *M. xanthus* cells expressing a McpM-GFP.
(AVI)
