# Supplementary figures and images for: Functional Organization of a Multimodular Bacterial Chemosensory Apparatus
Source: PLoS Genet. 2014 Mar 6;10(3):e1004164. doi: 10.1371/journal.pgen.1004164 (PMC3945109; doi:10.1371/journal.pgen.1004164)

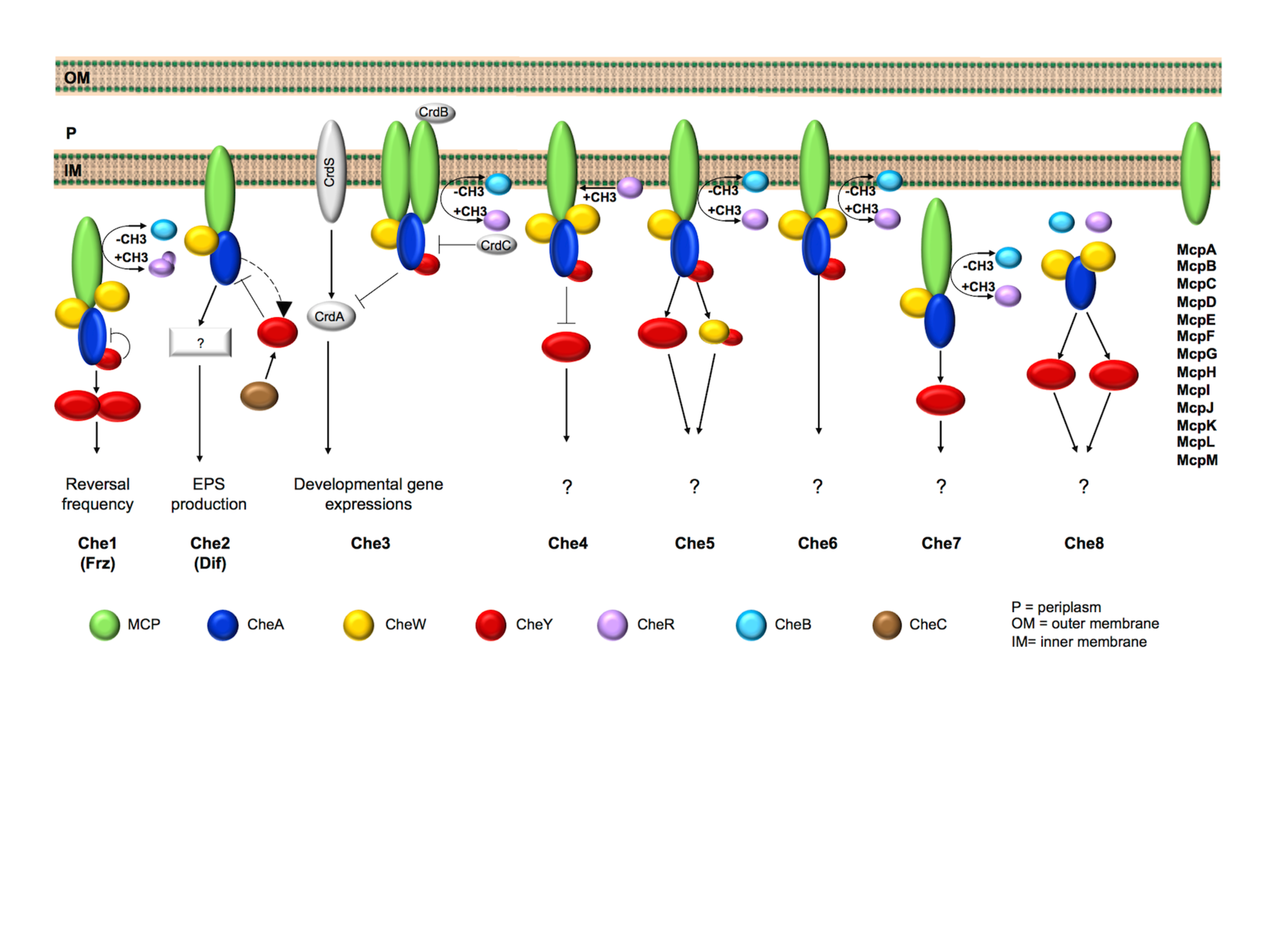

Supplement: Figure S1 — Schematic diagram of the putative organization of M. xanthus Che systems and orphan MCPs. Chemosensory proteins might form complexes analogously to their enteric counterparts. (TIF) [file pgen.1004164.s001.tif]

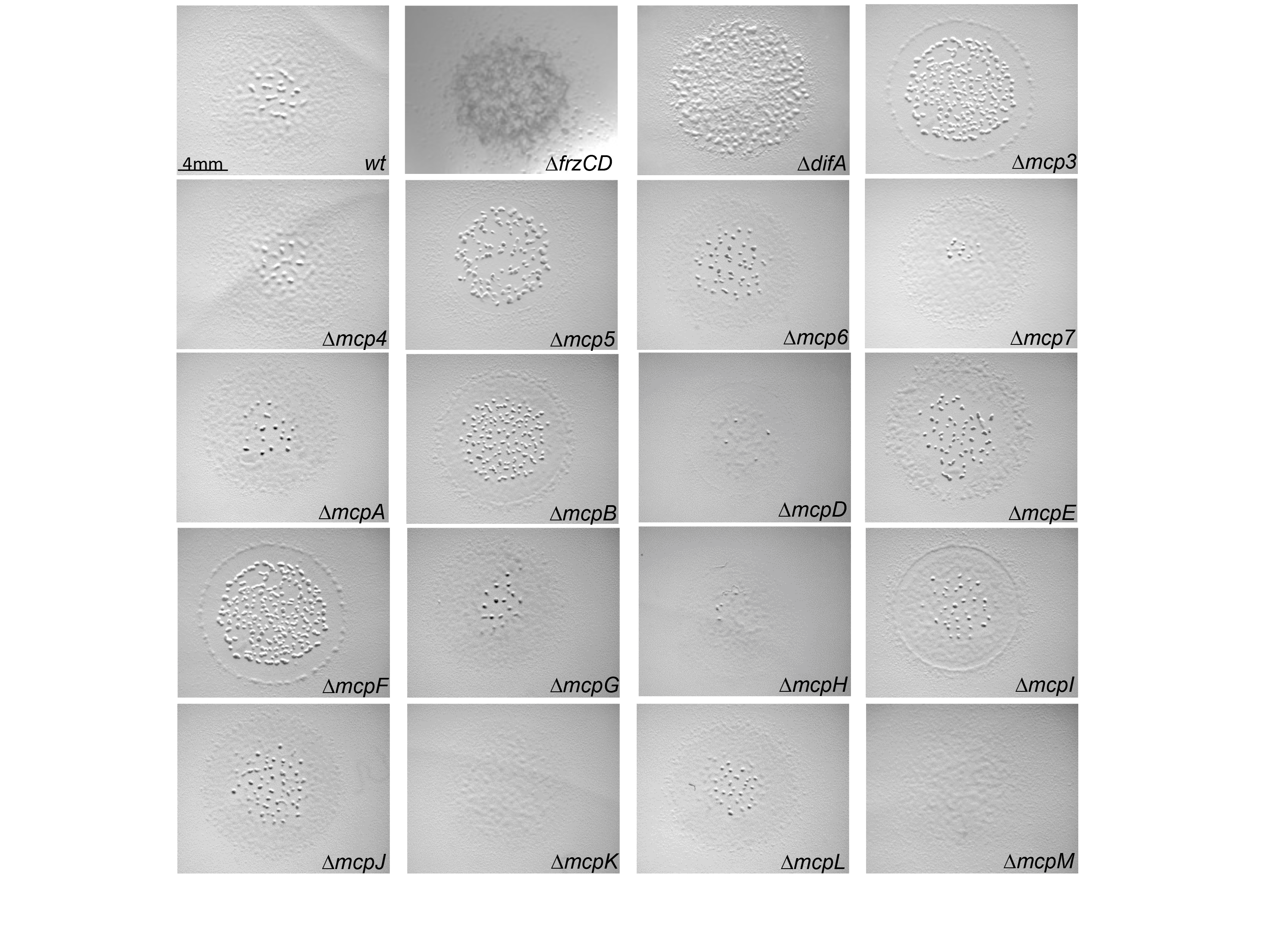

Supplement: Figure S2 — Fruiting body formation phenotypes of Δmcp mutants. Cells (5 µl), at a concentration of 4×10^9^ cfu ml−1, were spotted on CF plates containing an agar concentration of 1.5%, incubated at 32°C and photographed after 24, 48 and 72 h with a Olympus SZ61 microscope. Pictures taken at 48 h are shown. (TIF) [file pgen.1004164.s002.tif]

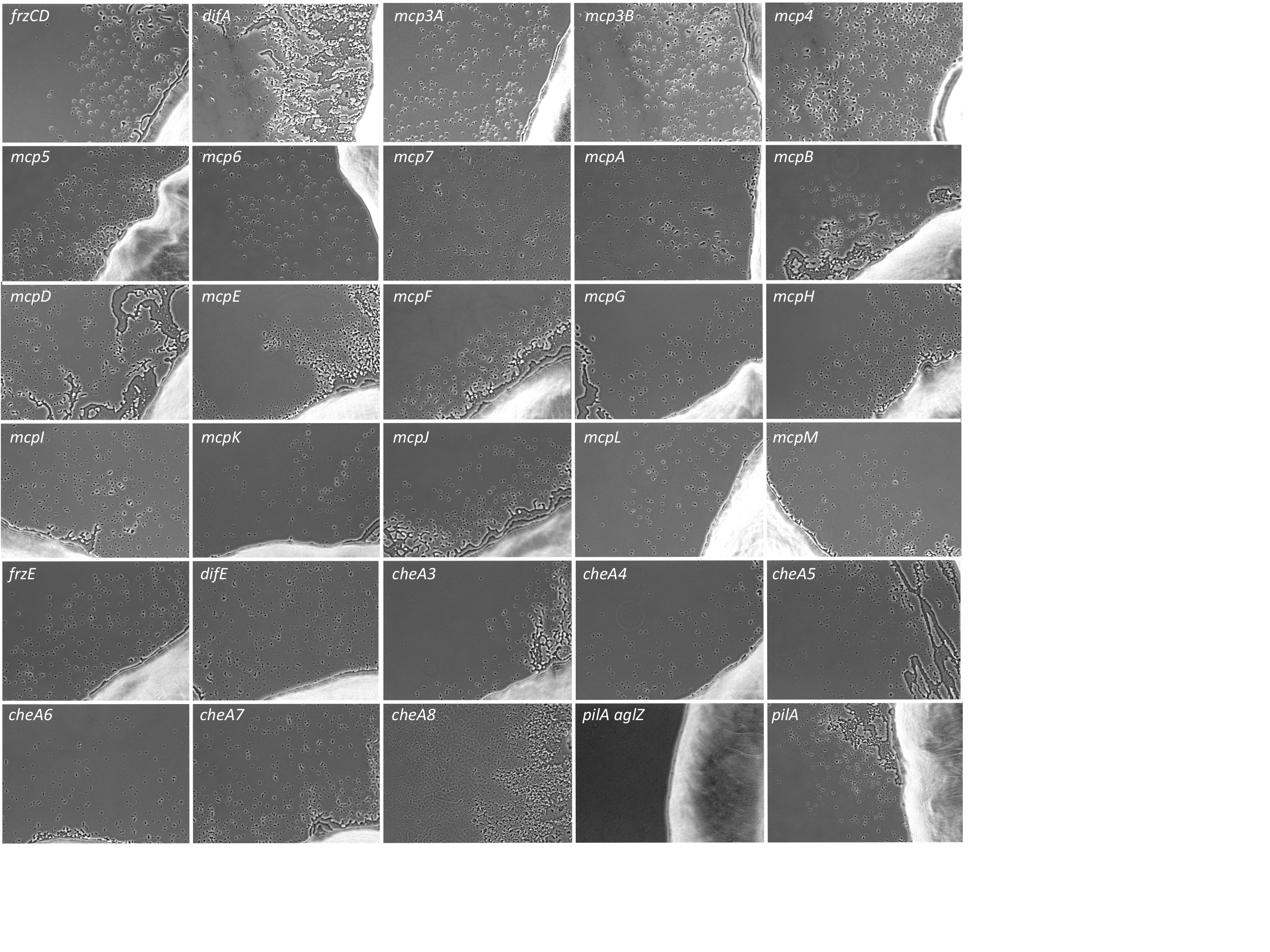

Supplement: Figure S3 — A-motility phenotypes of Δmcp/ΔcheA, pilA::tet double mutants. Cells (5 µl), at a concentration of 4×10^9^ cfu ml−1, were spotted on CYE plates containing an agar concentration of 1.5%, incubated at 32°C and the edge of each colony was photographed after 48 h with a 10x objective. (TIF) [file pgen.1004164.s003.tif]

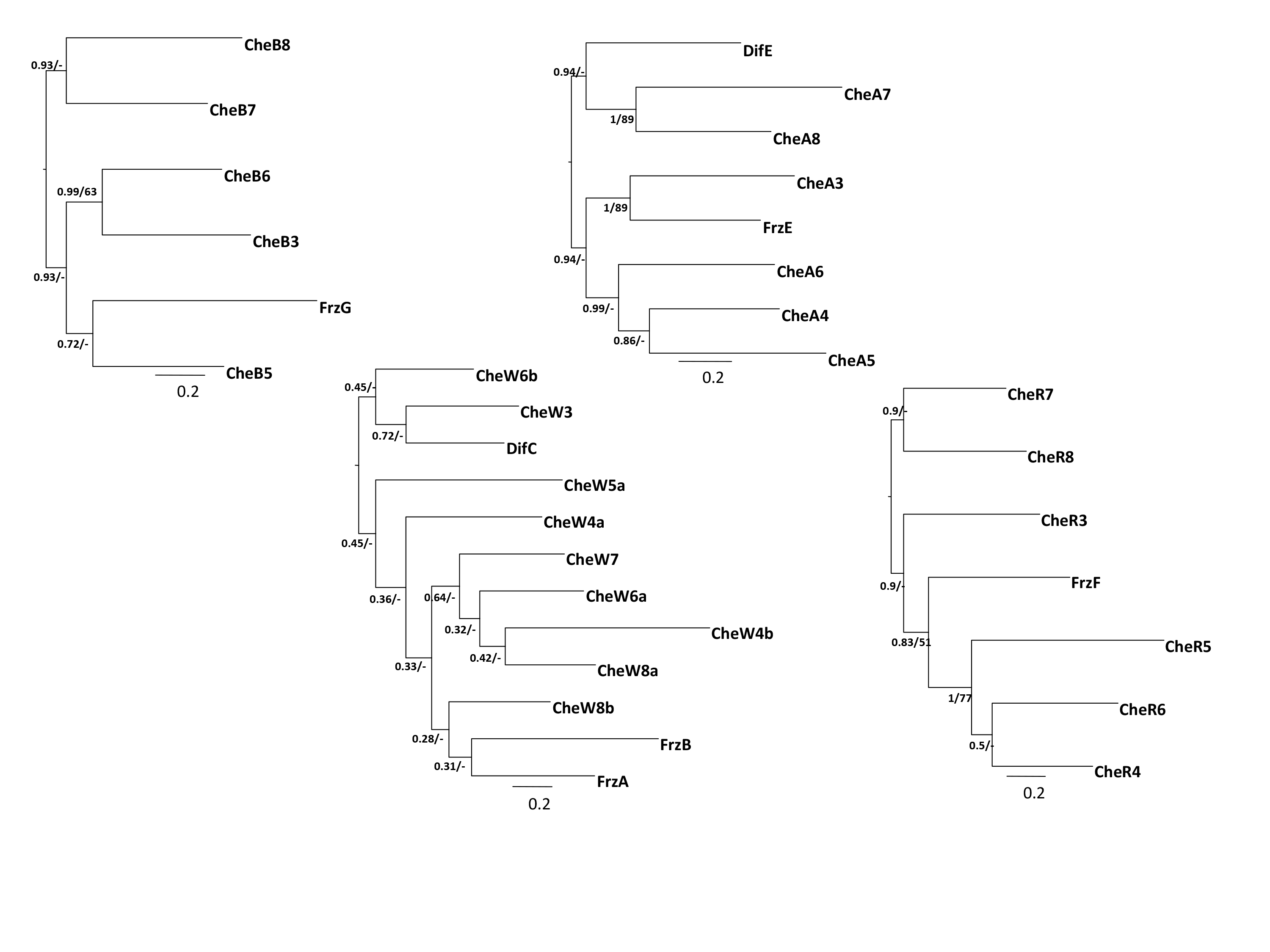

Supplement: Figure S4 — Phylogenetic relationships between M. xanthus Che homologues. Numbers at nodes indicate posterior probabilities (PP) computed by MrBayes and bootstrap values (BV) computed by PhyML. Only PP and BV above 0.5 and 50% are shown. The scale bars represent the average number of substitutions per site. (TIF) [file pgen.1004164.s004.tif]

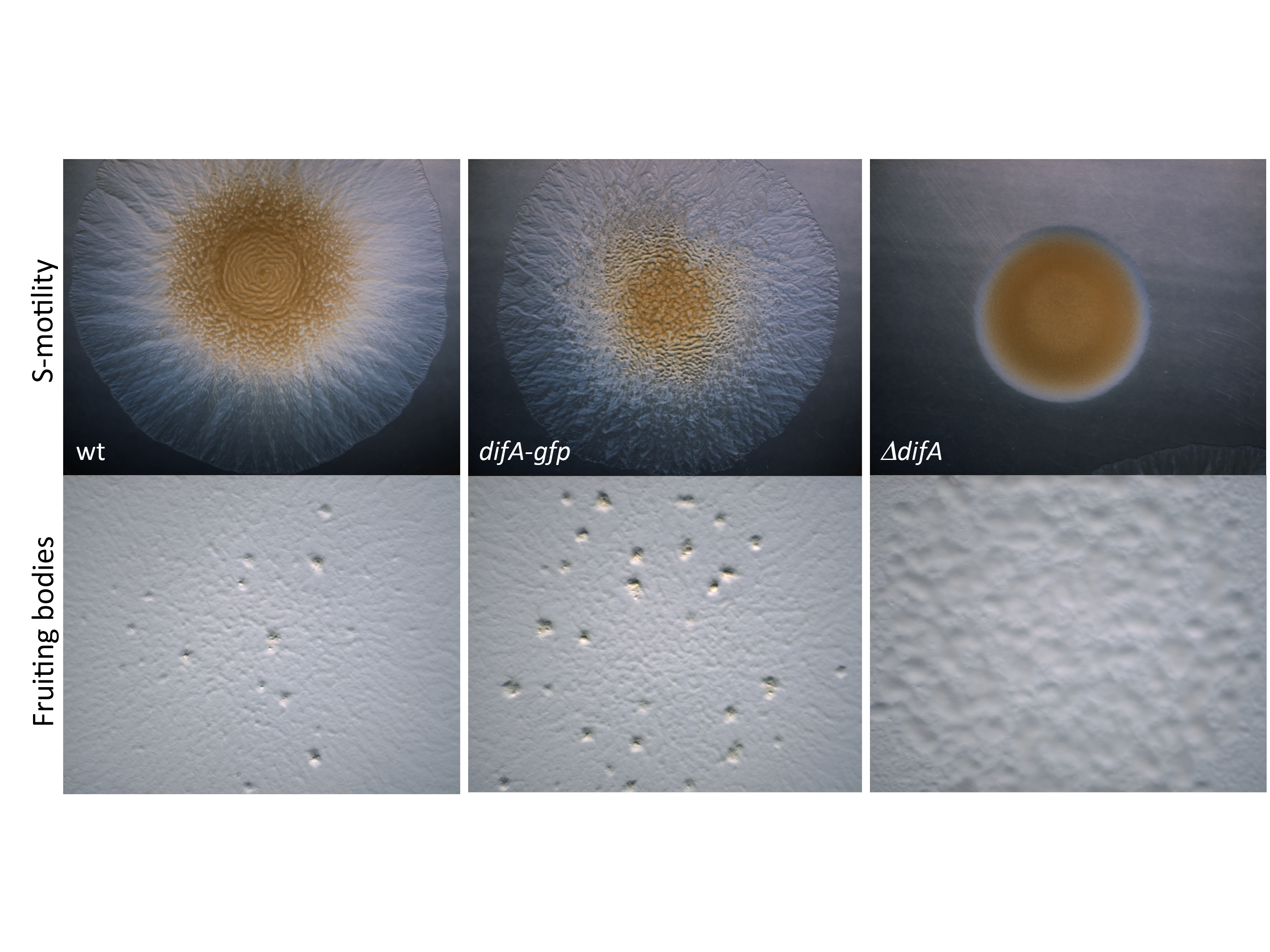

Supplement: Figure S5 — S motility and fruiting body formation phenotypes of a difA-gfp strain. Cells (5 µl), at a concentration of 4×10^9^ cfu ml−1, were spotted on CF-agar plates or CYE plates containing an agar concentration of 1.5% or 0.5%, respectively, incubated at 32°C and photographed after 48 or 72 h with a Olympus SZ61 microscope. (TIF) [file pgen.1004164.s005.tif]

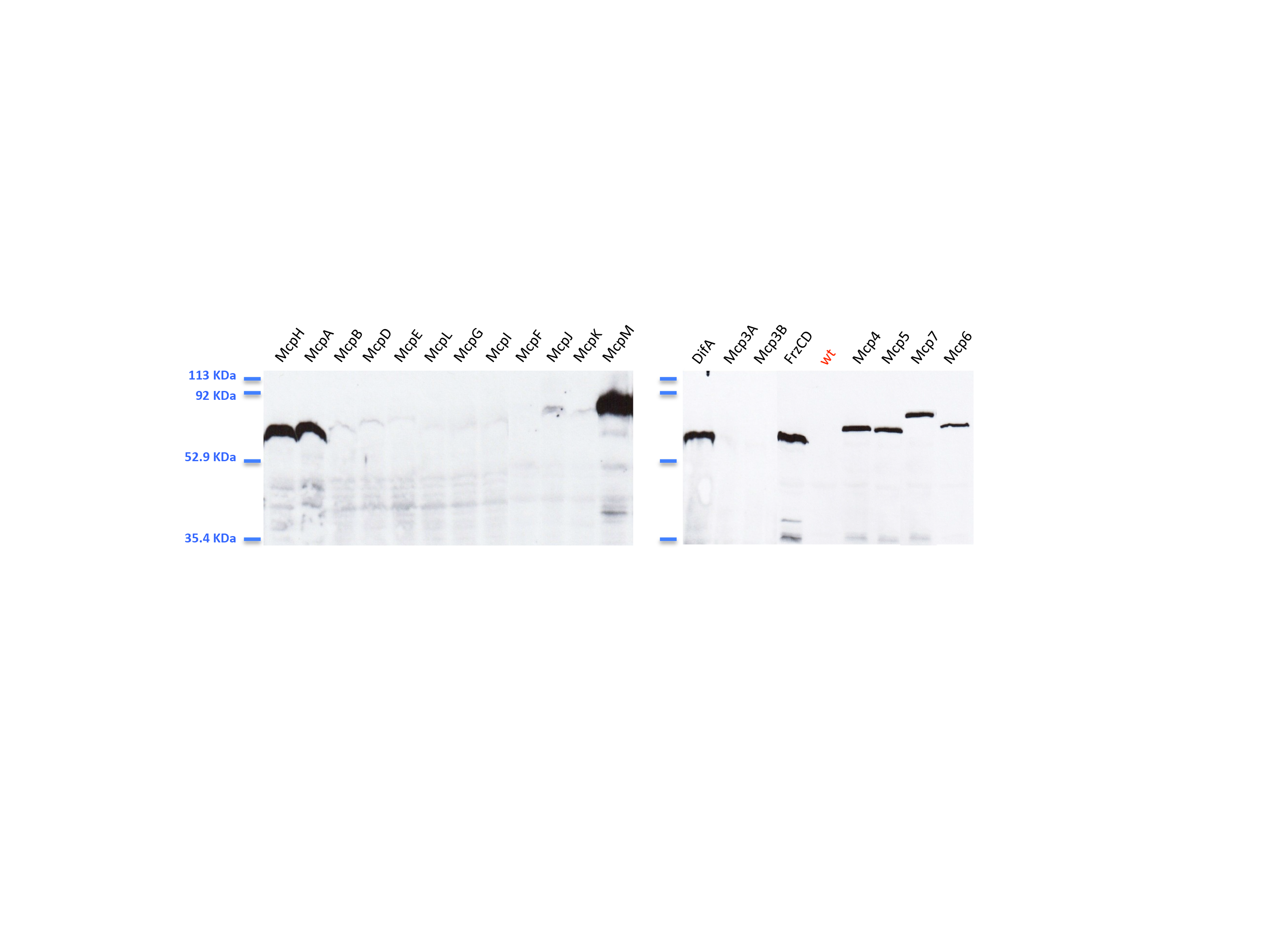

Supplement: Figure S6 — Mcp-GFP fusions are expressed in cells. Western blots using antibodies against GFP (Invitrogen) show stable expression of Mcp-GFP chimeras. Whole cell extracts were prepared from cells grown in liquid CYE media to mid-log phase. Ten micrograms of total proteins were loaded into 10% SDS-polyacrylammide gels. Proteins were then transferred to nitrocellulose membrane and immonoblots were prepared as previously described [50]. (TIF) [file pgen.1004164.s006.tif]

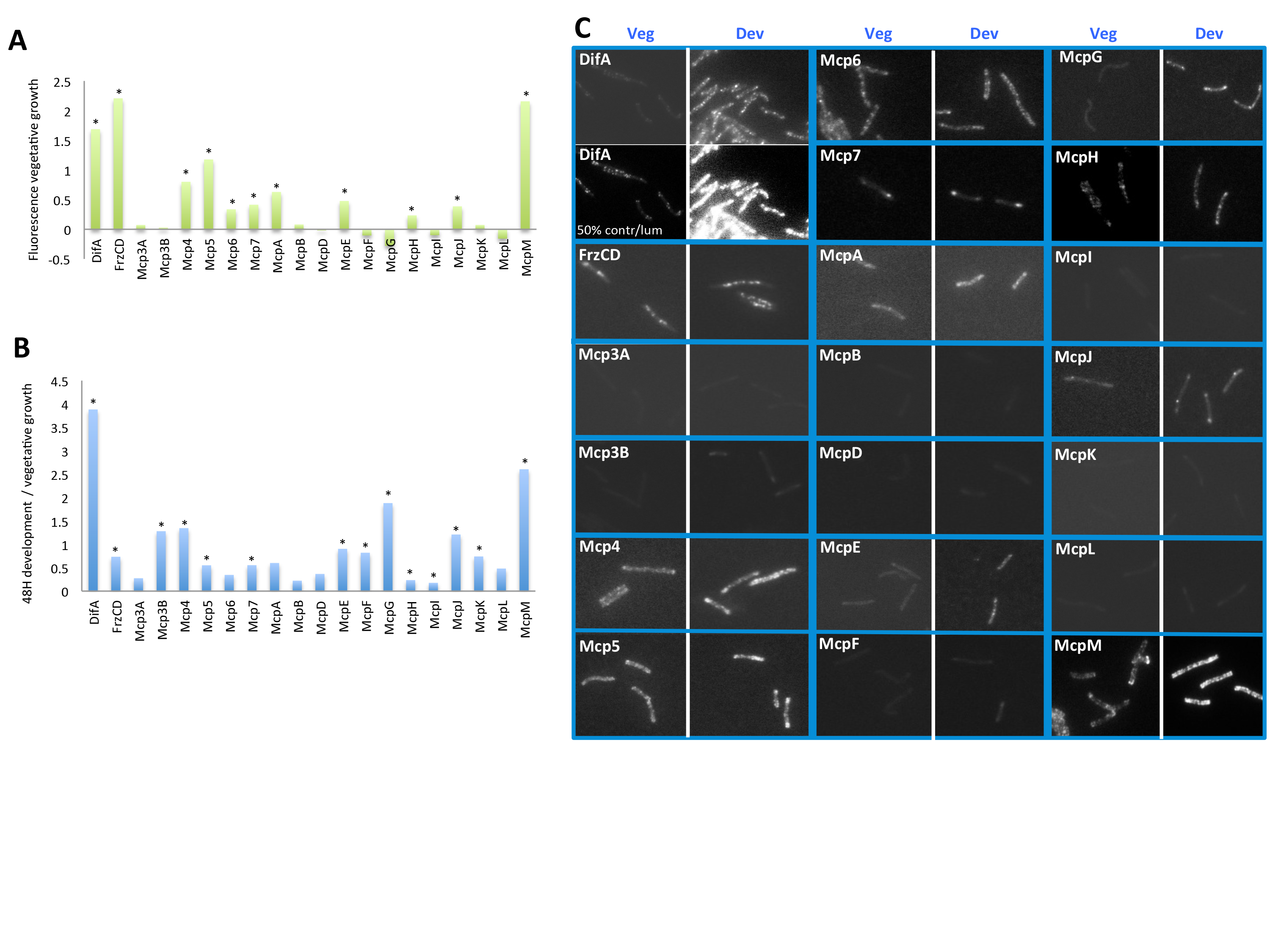

Supplement: Figure S7 — Cells expressing MCP-GFP proteins were grown in rich medium up to OD600 = 0.5 or incubated in CF medium in submerged cultures for 48 h as described by Kuner and Kaiser [67]. Cells were then collected and imaged at the fluorescence microscope as described in Material and Methods. The fluorescence intensity of approximately 200 cells per strain was measured with Fiji. We show (A) the fluorescence intensity from vegetative sample and (B) the ratio between the fluorescence intensity from developmental and vegetative samples. All values were normalized with the fluorescence intensity of wildtype samples. T tests were used to verify that the fluorescence intensity of the mcp-gfp strains was significant as compared to wt (* = p<5E-04). Examples of fluorescence images are shown in (C). (TIF) [file pgen.1004164.s007.tif]

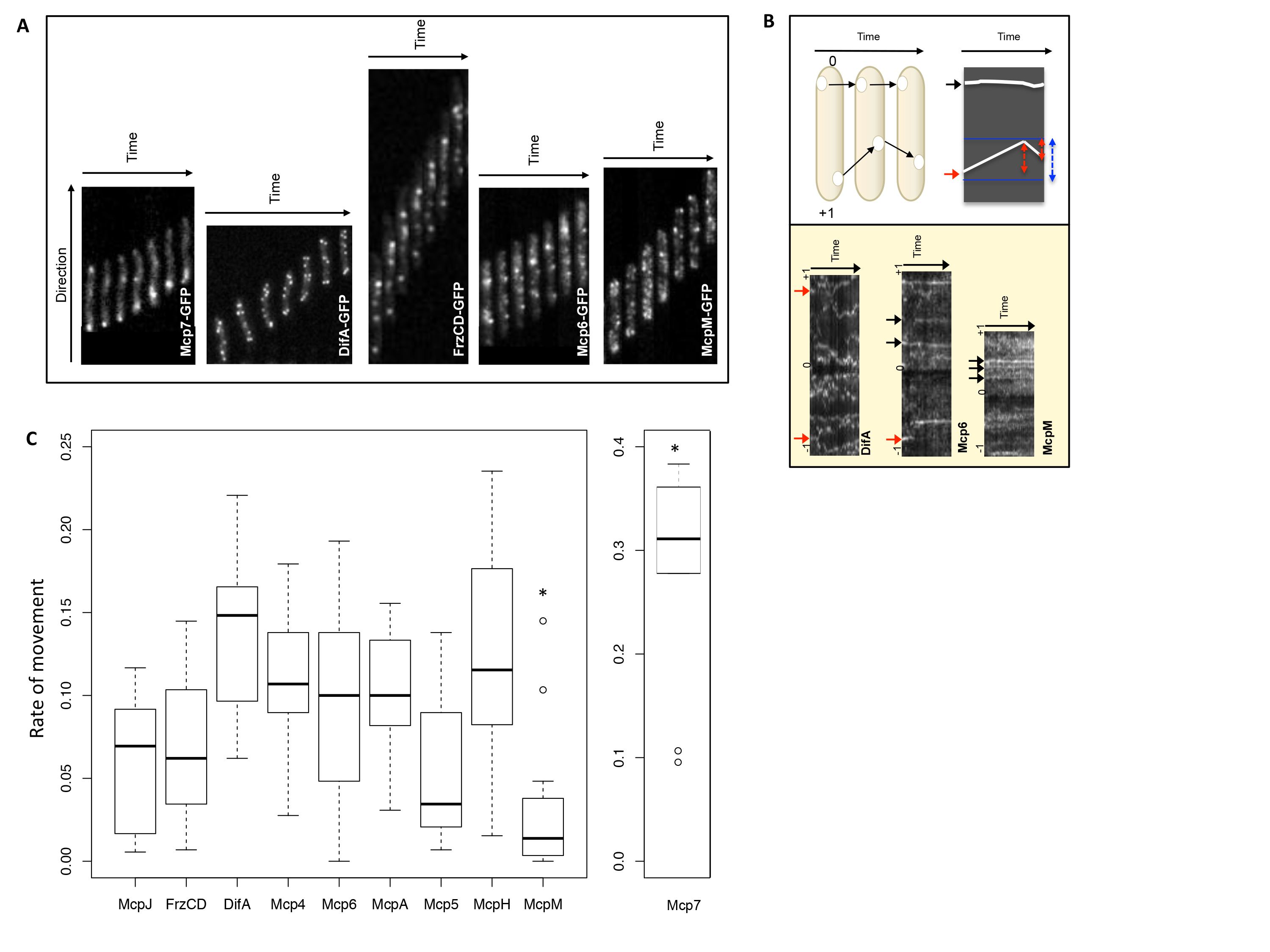

Supplement: Figure S8 — Mcp clusters are dynamic. (A) Time-lapse fluorescence microscopy of single cells imaged every 30 seconds. (B) Kimographs obtained from time-lapse fluorescence microscopy of single cells imaged every 5 seconds. For the cytoplasmic FrzCD-GFP, kimographs were realized by analyzing cells from one pole (−1) to the other (0). For the remaining trasmembrane MCP-GFP fusions, kimographs were realized by analyzing the whole perimeter of cells from one pole (−1) to the other (0) and, then, returning to the first pole (+1). Red and black arrows indicate static and dynamic clusters, respectively. (C) Box plots indicate the medians of the distance covered in a given time by the MCP-GFP clusters. * = p<5E-04 (See also Methods and Table S2). (TIF) [file pgen.1004164.s008.tif]

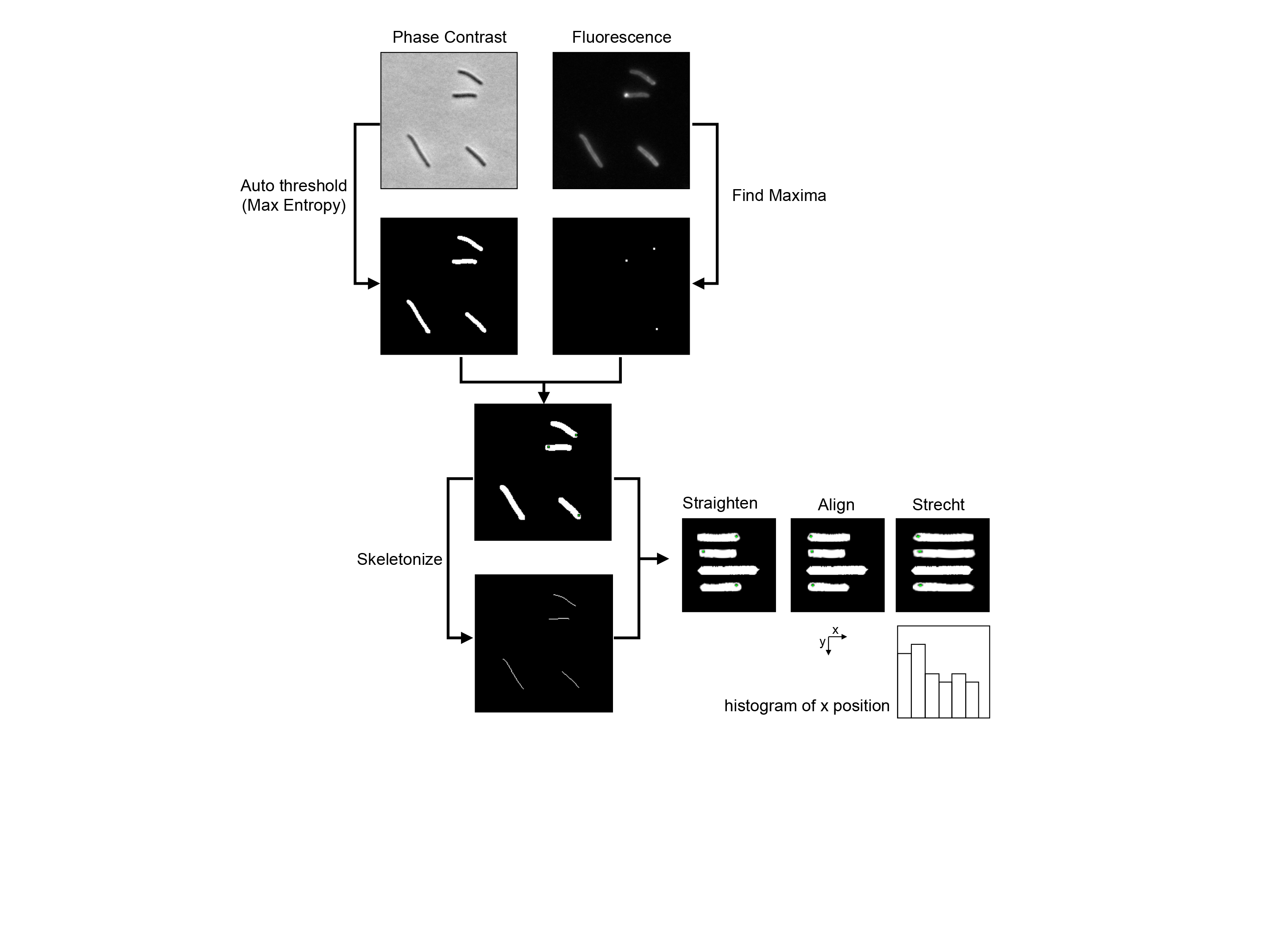

Supplement: Figure S9 — Image J based work flow used to detect MCP-GFP clusters. (TIF) [file pgen.1004164.s009.tif]
