# Supplementary material for: Functional Organization of a Multimodular Bacterial Chemosensory Apparatus
Source: PLoS Genet. 2014 Mar 6;10(3):e1004164. doi: 10.1371/journal.pgen.1004164 (PMC3945109; doi:10.1371/journal.pgen.1004164)
Supplement: Table S1 — Strains and plasmids. (PDF) [file pgen.1004164.s010.pdf]

Table S1. Strains and Plasmids

Plasmid	Expression plasmid	Source
pEM168	pBJ113 with a cassette for *difA-gfp*	This study
pEM169	pBJ113 with a cassette for *mcp3A-gfp*	This study
pEM170	pBJ113 with a cassette for *mcp3B-gfp*	This study
pEM172	pBJ113 with a cassette for *mcpJ-gfp*	This study
pEM173	pBJ113 with a cassette for *mcp7-gfp*	This study
pEM178	pBJ113 with a cassette for *mcp4-gfp*	This study
pEM177	pBJ113 with a cassette for *mcpE-gfp*	This study
pEM180	pBJ113 with a cassette for *mcp6-gfp*	This study
pEM291	pBJ113 with a cassette for *mcp5-gfp*	This study
pEM292	pBJ113 with a cassette for *mcpA-gfp*	This study
pEM293	pBJ113 with a cassette for *mcpB-gfp*	This study
pEM294	pBJ113 with a cassette for *mcpD-gfp*	This study
pEM295	pBJ113 with a cassette for *mcpF-gfp*	This study
pEM296	pBJ113 with a cassette for *mcpG-gfp*	This study
pEM297	pBJ113 with a cassette for *mcpH-gfp*	This study
pEM298	pBJ113 with a cassette for *mcpI-gfp*	This study
pEM299	pBJ113 with a cassette for *mcpK-gfp*	This study
pEM300	pBJ113 with a cassette for *mcpL-gfp*	This study
pEM301	pBJ113 with a cassette for *mcpM-gfp*	This study
pEM394	pBJ113 with a cassette for *mcp5-mCherry*	This study
pEM395	pBJ113 with a cassette for *mcp6-mCherry*	This study
pEM255	pBJ113 with a deletion cassette for *mcpB*	This study
pEM256	pBJ113 with a deletion cassette for *mcpE*	This study
pEM261	pBJ113 with a deletion cassette for *mcpG*	This study
pEM262	pBJ113 with a deletion cassette for *mcpJ*	This study
pEM263	pBJ113 with a deletion cassette for *mcpK*	This study
pEM264	pBJ113 with a deletion cassette for *mcpL*	This study
pEM265	pBJ113 with a deletion cassette for *mcpM*	This study
pEM266	pBJ113 with a deletion cassette for *mcp5*	This study
pEM267	pBJ113 with a deletion cassette for *mcpD*	This study
pEM268	pBJ113 with a deletion cassette for *mcpF*	This study
pEM269	pBJ113 with a deletion cassette for *mcpM*	This study
pEM270	pBJ113 with a deletion cassette for *mcpI*	This study
pEM271	pBJ113 with a deletion cassette for *mcp6*	This study
pEM272	pBJ113 with a deletion cassette for *mcp7*	This study
pEM275	pBJ113 with a deletion cassette for *mcpA*	This study
pEM278	pBJ113 with a deletion cassette for *difA*	This study
pEM318	pBJ113 with a deletion cassette for *cheA8*	This study
pEM319	pBJ113 with a deletion cassette for *cheA3*	This study
pEM320	pBJ113 with a deletion cassette for *cheA5*	This study
pEM321	pBJ113 with a deletion cassette for *cheA6*	This study
pEM322	pBJ113 with a deletion cassette for *cheA7*	This study
pEM323	pBJ113 with a deletion cassette for *cheA4*	This study

Strain	Genotype	Deletion	Source
DZ2	*wt*		Zusman et al., 1982
DZ4620	*frzCD-gfp*		Mauriello et al., 2009
EM248	*mcpA-gfp*		This study
EM256	*mcpL-gfp*		This study
EM258	*mcp5-gfp*		This study
EM273	*mcp3A-gfp*		This study
EM274	*mcp3B-gfp*		This study
EM275	*difA-gfp*		This study
EM276	*mcpJ-gfp*		This study
EM277	*mcp7-gfp*		This study
EM281	*mcpE-gfp*		This study
EM282	*mcp4-gfp*		This study
EM285	*mcp6-gfp*		This study
EM403	*mcpK-gfp*		This study
EM404	*mcpB-gfp*		This study
EM405	*mcpH-gfp*		This study
EM407	*mcpG-gfp*		This study
EM408	*mcpI-gfp*		This study
EM409	*mcpD-gfp*		This study
EM410	*mcpM-gfp*		This study
EM411	*mcpF-gfp*		This study
EM501	*mcp5-mCherry, mcpM-gfp*		This study
EM502	*mcp5-mCherry, mcp6-gfp*		This study
EM503	*mcp5-mCherry, mcpH-gfp*		This study
EM507	*mcp6-mCherry, mcp4-gfp*		This study
EM508	*mcp6-mCherry, mcpA-gfp*		This study
DZ4734	*frzCD-gfp, aglZ-mCherry::kan*		Mauriello et al., 2009
EM360	Δ*mcpJ*	codons 101–707	This study
EM361	Δ*mcpB*	codons 108–448	This study
EM362	Δ*mcpE*	codons 108–507	This study
EM363	Δ*mcpL*	codons 108–585	This study
EM364	Δ*mcp5*	codons 8–519	This study
EM365	Δ*mcpI*	codons 9–495	This study
EM366	Δ*mcp6*	codons 11–527	This study
EM369	Δ*mcp7*	codons 9–801	This study
EM370	Δ*mcpD*	codons 10–487	This study
EM371	Δ*mcpG*	codons 112–441	This study
EM372	Δ*mcpH*	codons 101–707	This study
EM373	Δ*mcpK*	codons 119–939	This study
EM374	Δ*mcpM*	codons 127–622	This study
EM375	Δ*mcpF*	codons 10–719	This study
EM378	Δ*mcpA*	codons 10–453	This study

EM379	Δ*difA*	codons 9–402	This study
DZ4480	Δ*frzCD*	codons 6–393	Bustamante et al ; 2004
DZ4504	Δ*mcp3A*	codons 8–576	Kirby et al ; 2003
DZ4506	Δ*mcp3B*	codons 8–588	Kirby et al ; 2003
DZ4451	Δ*mcp4*	codons 34–797	Vlamikis et al ; 2004
DZ4481	Δ*frzE*	codons 13–766	Bustamante et al., 2004
EM450	Δ*difE*	codons 12-770	This study
EM415	Δ*cheA7*	codons 16-651	This study
EM416	Δ*cheA6*	codons 20-708	This study
EM417	Δ*cheA4*	codons 15-822	This study
EM420	Δ*cheA3*	codons 16-701	This study
EM421	Δ*cheA5*	codons 9-687	This study
EM432	Δ*cheA8*	codons 3-851	This study
EM446	Δ*cheA4*, Δ*cheA6*		This study
EM454	Δ*cheA4*, Δ*cheA5*		This study
EM460	Δ*cheA5*, Δ*cheA6*		This study
EM494	Δ*cheA4*, Δ*cheA5*, Δ*cheA6*		This study
EM207	Δ*frzCD pilA::tet*		This study
EM463	Δ*difA pilA::tet*		This study
EM464	Δ*mcp3A pilA::tet*		This study
EM465	Δ*mcp3B pilA::tet*		This study
EM466	Δ*mcp4 pilA::tet*		This study
EM467	Δ*mcp5 pilA::tet*		This study
EM468	Δ*mcp6 pilA::tet*		This study
EM469	Δ*mcp7 pilA::tet*		This study
EM470	Δ*mcpA pilA::tet*		This study
EM471	Δ*mcpB pilA::tet*		This study
EM472	Δ*mcpD pilA::tet*		This study
EM473	Δ*mcpE pilA::tet*		This study
EM474	Δ*mcpF pilA::tet*		This study
EM475	Δ*mcpG pilA::tet*		This study
EM476	Δ*mcpH pilA::tet*		This study
EM477	Δ*mcpI pilA::tet*		This study
EM478	Δ*mcpL pilA::tet*		This study
EM479	Δ*mcpM pilA::tet*		This study
EM480	Δ*mcpJ pilA::tet*		This study
EM481	Δ*mcpK pilA::tet*		This study
EM482	Δ*difE pilA::tet*		This study
EM206	Δ*frzE pilA::tet*		This study
EM483	Δ*cheA3 pilA::tet*		This study
EM484	Δ*cheA4 pilA::tet*		This study
EM485	Δ*cheA5 pilA::tet*		This study
EM486	Δ*cheA6 pilA::tet*		This study
EM487	Δ*cheA7 pilA::tet*		This study
EM488	Δ*cheA8 pilA::tet*		This study
