# Supplementary material for: Functional Organization of a Multimodular Bacterial Chemosensory Apparatus
Source: PLoS Genet. 2014 Mar 6;10(3):e1004164. doi: 10.1371/journal.pgen.1004164 (PMC3945109; doi:10.1371/journal.pgen.1004164)
Supplement: Table S2 — T values (Wilcoxon tests). (PDF) [file pgen.1004164.s011.pdf]

Table S2. Tvalues (Wilcoxon tests)

Covered length * total displacement	McpJ	Mcp7	FrzCD	DifA	Mcp4	Mcp6	McpA	Mcp5	McpH	McpM
McpJ										
Mcp7	0.0002041									
FrzCD	0.415	0.000008108								
DifA	0.8841	0.000008108	0.02236							
Mcp4	0.7045	0.00001713	0.135	0.2505						
Mcp6	0.05966	0.000006804	0.2486	0.0003636	0.00448					
McpA	0.0276	4.267E-07	0.01557	7.696E-07	0.00007175	0.3048				
Mcp5	0.01398	0.00004188	0.01671	0.00002572	0.0002406	0.2779	0.8045			
McpH	0.01077	0.000002447	0.006961	4.115E-07	0.00001356	0.106	0.4195	0.648		
McpM	0.001448	0.00004982	0.0003514	0.00001068	0.00009958	0.0187	0.01229	0.05835	0.064	

Rate of movement	McpJ	Mcp7	FrzCD	DifA	Mcp4	Mcp6	McpA	Mcp5	McpH	McpM
McpJ										
Mcp7	0.0004277									
FrzCD	0.5112	0.0002308								
DifA	0.0001755	0.004987	0.0001029							
Mcp4	0.009344	0.002756	0.01923	0.07364						
Mcp6	0.1121	0.002517	0.2435	0.03732	0.5536					
McpA	0.02093	0.0008984	0.04782	0.01412	0.6347	0.8489				
Mcp5	0.6411	0.00007903	0.1118	0.00001086	0.001084	0.03309	0.001005			
McpH	0.01913	0.003062	0.0387	0.518	0.7303	0.3158	0.4471	0.00505		
McpM	0.04293	0.0001002	0.002897	0.000006391	0.0001626	0.007839	0.0001546	0.1108	0.0002685	
